# Pentose phosphate pathway inhibition metabolically reprograms CD8^+^ T cells and disrupts CNS autoimmunity

**DOI:** 10.1172/jci.insight.184240

**Published:** 2025-06-10

**Authors:** Ethan M. Grund, Benjamin D.S. Clarkson, Susanna Pucci, Maria S. Westphal, Carolina Muniz Partida, Sara A. Muhammad, Charles L. Howe

**Affiliations:** 1 Mayo Graduate School,; 2 Mayo Clinic Medical Scientist Training Program,; 3 Translational Neuroimmunology Laboratory,; 4 Department of Laboratory Medicine and Pathology,; 5 Center for Multiple Sclerosis and Autoimmune Neurology,; 6 Department of Neurology, and; 7 Division of Experimental Neurology, Mayo Clinic, Rochester, Minnesota, USA.

**Keywords:** Autoimmunity, Immunology, Neuroscience, Autoimmune diseases, Glucose metabolism, Multiple sclerosis

## Abstract

Multiple sclerosis is characterized by CNS infiltration of autoreactive immune cells that drive both acute inflammatory demyelination and chronic progressive axonal and neuronal injury. Expanding evidence implicates CD8^+^ antineural T cells in the neurodegeneration that underlies irreversible clinical progression in multiple sclerosis, yet therapies specifically targeting this cell population are limited. CD8^+^ T cells from patients with MS exhibit increased engagement of the pentose phosphate pathway. Pharmacologic inhibition of the pentose phosphate pathway reduced glycolysis, glucose uptake, NADPH production, ATP production, proliferation, and proinflammatory cytokine secretion in CD8^+^ T cells activated by ligation of CD3 and CD28. Pentose phosphate pathway inhibition also prevented CD8^+^ T cell–mediated antigen-specific neuronal injury in vitro and in both an adoptive transfer–based cuprizone model of demyelination and in mice with experimental autoimmune encephalomyelitis. Notably, transcriptional profiling of CNS-infiltrating CD8^+^ T cells in patients with MS indicated increased pentose phosphate pathway engagement, suggesting that this pathway is involved in CD8^+^ T cell–mediated injury of axons and neurons in the demyelinated CNS. Inhibiting the pentose phosphate pathway disrupts CD8^+^ T cell metabolic reprogramming and effector functions, suggesting that such inhibition may serve as a therapeutic strategy to prevent neurodegeneration in patients with progressive MS.

## Abstract 2

Inhibiting the pentose phosphate pathway in autoimmune CD8^+^ T cells reduces their neurotoxic effects, offering a potential therapeutic strategy for progressive multiple sclerosis.

## Graphical abstract

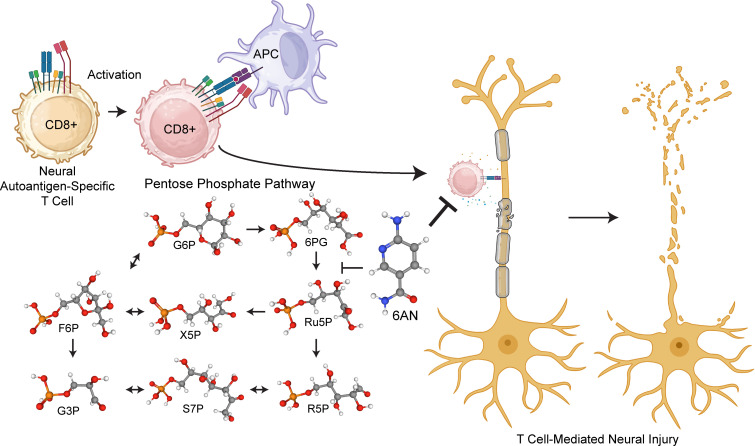

## Introduction

Multiple sclerosis (MS) affects nearly 3 million people and is the leading cause of nontraumatic neurologic deficits in young adults. CD8^+^ T cell clones are more highly enriched than CD4^+^ T cells in the blood (1) and cerebrospinal fluid (CSF) (2) of patients with MS and outnumber CD4^+^ T cells by a factor of 10 in active demyelinating lesions. Oligoclonal populations of T lymphocytes have been identified in blood and CSF of patients with MS, suggesting antigen-directed expansion of disease-related T cell clonotypes (3). Histopathologically, clusters of CD8^+^ T cells are observed adjoining degenerating axons, and prevalence of these cells correlates with the extent of ongoing axonal injury within demyelinated lesions (4). We have previously demonstrated that demyelination induces expression of MHC class I on neurons and axons and results in presentation of neuron-specific antigens to surveilling antigen-specific CD8^+^ T cells, resulting in proliferation, CNS accumulation, and neuronal injury (5). These findings highlight cytotoxic CD8^+^ T cells as potential mediators of axonal loss in the demyelinated CNS (6–8) and suggest that these cells may be promising therapeutic targets to specifically prevent neurodegeneration and secondary brain atrophy in patients with progressive MS (9, 10).

Metabolic reprogramming, conserved across diverse antigen-driven stimuli, is essential for T cell differentiation and effector function. Targeting immunometabolism may, therefore, provide an opportunity to develop antigen-agnostic autoimmune interventions. Biochemical pathway reorganization dictates many functional aspects of immunobiology (11–13), as proinflammatory immune cells undergo a dramatic upregulation of biosynthetic pathways (14, 15) upon interaction with cognate antigens or proinflammatory stimuli (16). Activated T cells rapidly shift to anabolic glycolysis (Warburg effect) to fuel maximal CD8^+^ T cell effector functions (16–19). This shift involves hyperacute activation of pyruvate dehydrogenase kinase 1, which shunts glucose-derived intermediates toward lactic acid production, uncoupling cytosolic glucose metabolism from mitochondrial oxidative phosphorylation (20). Stable isotope labeling studies have shown that, following TCR ligation, glucose is reallocated from glycolysis toward increased engagement of the pentose phosphate pathway (PPP) (21). The PPP is a biochemical shunt from early glycolysis that supports redox balancing, cholesterol/lipid biosynthesis through NADPH production, and purine biosynthesis for DNA replication. At present, whether increased PPP engagement is essential for the differentiation and effector functions of CD8^+^ T cells in human autoaggressive diseases remains unknown.

Immunometabolic interventions have proven effective in proinflammatory autoaggressive diseases such as MS (22, 23). However, therapies targeting the PPP in MS have not been evaluated. Current therapies for MS that are thought to act by modulating metabolic reprogramming in immune cells include teriflunomide and dimethyl fumarate (24). These therapies have demonstrated efficacy in disrupting proliferation and proinflammatory metabolism within lymphocytes to improve outcomes in autoaggressive diseases such as rheumatoid arthritis and have been shown to reduce clinical relapses in patients with MS. Mechanistically, this is attributed to the ability of these agents to prevent T cells from undergoing metabolic shifts necessary to meet the bioenergetic and redox biology demands necessary for maximal immune activation (25). Given the metabolic adaptations downstream of TCR signaling that converge to increase PPP engagement, we sought to determine whether PPP engagement is increased in autoaggressive disease states such as MS and if inhibition of the PPP would disrupt cytotoxic effector functions to ameliorate CD8^+^ T cell–mediated axonal injury in the demyelinated CNS.

## Results

### CD8^+^ T cells engage the PPP under conditions of inflammatory demyelination.

To confirm that CD8^+^ T cells upregulate anabolic glycolysis upon immune activation, we isolated murine splenocytes (Supplemental Figure 1A; supplemental material available online with this article; https://doi.org/10.1172/jci.insight.184240DS1) and ligated CD3 and CD28 with plate-bound activating antibodies (hereafter termed CD3/28), simulating antigen driven T cell activation. By flow cytometry, lymphocytes stimulated with plate-bound CD3/28 for 24 hours demonstrate increased uptake of the glucose analog 2-NBDG compared with unstimulated controls (UNT) (Supplemental Figure 1B). By serially measuring the extracellular acidification rate (ECAR) as an indicator of glycolysis-driven lactic acid production, we observed that immunomagnetically enriched CD8^+^ lymphocytes stimulated by CD3/28 ligation exhibited elevated ECAR and possessed greater glycolytic capacity (Supplemental Figure 1C; CD8-enriched CD3/28 versus CD8-depleted CD3/28, *P* = 0.0029) compared with CD8-depleted splenocytes, consistent with engagement of Warburg metabolism. To rule out an effect of differential cell survival on the metabolic readouts, we assessed cell death by flow cytometry. We did not observe a difference between unstimulated and stimulated T cells (Supplemental Figure 1D).

**Figure 1:**
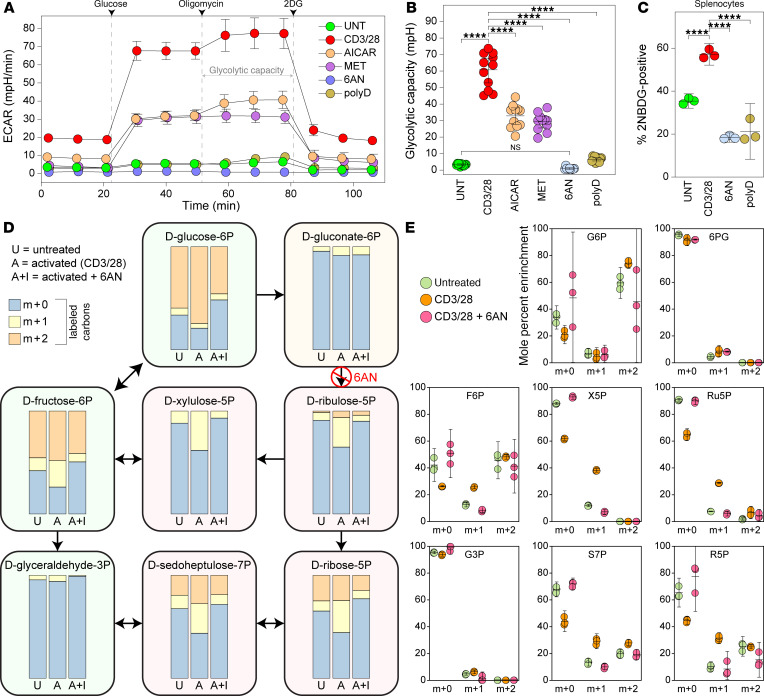
Activated CD8^+^ T cells engage the PPP. (**A**) Realtime extracellular acidification rate (ECAR) was measured using the Seahorse XF analyzer in unstimulated (UNT) or CD3/28-activated (CD3/28) CD8^+^ splenocytes in the presence of the AMPK activators AICAR (500 μM) and metformin (MET) (10 mM) or the PPP inhibitors 6AN (1 mM) and polydatin (polyD) (1 mM). (**B**) Glycolytic capacity derived from the ECAR in unstimulated (UNT) or CD3/28-activated (CD3/28) CD8^+^ splenocytes in the presence of AICAR, MET, 6AN, or polyD. *n* = 11 technical replicates/condition. (**C**) Glucose uptake evaluated at 24 hours in UNT or CD3/28-activated splenocytes inhibited with 6AN (1 mM) or polyD (1 mM); *n* = 3. (**D**) Summary representation of isotope distribution in PPP pathway molecules in CD8^+^ splenocytes 4 hours after the addition of D-glucose-1,2-^13^C_2_. Graphs show mole percent enrichment for the m+0, m+1, and m+2 species in cells that were untreated (U), activated with CD3/28 (A), or activated with CD3/28 in the presence of 6AN (100 μM) (A+I). (**E**) Individual mole percent enrichment data for each species in each treatment condition (*n* = 3 per condition) shown in **D**. Due to the density of data, individual statistics are provided in Supplemental Table 1. One-way or 2-way ANOVA with Tukey’s or Dunnet’s pairwise comparison test was used to assess significance. ****P < 0.0001. Data in **A**–**C** are representative of at least 3 separate experiments. Data are shown as mean ± 95% CI.

To broadly assess other biochemical pathways necessary for induction of anaerobic glycolysis, we used a panel of immunometabolic inhibitors, delivered at the time of splenocyte activation. This pharmacologic approach demonstrated robust inhibition of extracellular acidification with the PPP inhibitors polydatin and 6-aminonicotinamide (6AN), which inhibit the NADPH-producing enzymes glucose-6-dehydrogenase (converts glucose-6-phosphate to 6-phosphogluconolactone) and 6-phosphogluconate dehydrogenase (converts 6-phosphogluconic acid to ribulose 5-phosphate), respectively. PPP inhibition resulted in suppression of ECAR to levels measured in unstimulated cells (Figure 1A). Quantification of glycolytic capacity in this assay revealed that PPP inhibition suppressed glycolysis following CD3/28 activation (Figure 1B, UNT versus CD3/28 *P* < 0.0001) to unstimulated levels (UNT versus 6AN *P* = 0.954, UNT versus polydatin *P* = 0.796). Additionally, incorporation of glucose as demonstrated by 2-NBDG uptake was decreased with PPP inhibition (Figure 1C; CD3/28 versus PPP inhibition, *P* < 0.0001), demonstrating suppression of increased glucose uptake in response to activation. Due to polydatin exhibiting greater toxicity to neurons relative to 6AN (data not shown), 6AN was used for PPP inhibition in all subsequent experiments. Cell viability assessed by flow cytometry indicated that 6AN did not significantly affect T cell survival at concentrations up to 200 μM (Supplemental Figure 1E; untreated versus 100 μM, unstimulated: *P* = 0.5156; untreated versus 100 μM, CD3/28 stimulated: *P* = 0.2356; by Tukey’s multiple comparison). Using stable carbon isotope tracing in cells fed D-glucose-1,2-^13^C_2_, we showed that CD3/28 stimulation strongly promoted glucose flux through the PPP pathway in immunomagnetically isolated CD8^+^ splenocytes, and this flux was inhibited by 6AN treatment (Figure 1, D and E, and Supplemental Table 1). Analysis of isotope incorporation into lactate and subsequent calculation of pentose cycle activity (PCA) (26) confirmed this observation (untreated PCA = 1.7 ± 0.3%; CD3/28 stimulated PCA = 2.7 ± 0.2%; CD3/28 + 6AN PCA = 1.3 ± 0.2%; F[2,6] = 29.04, *P* = 0.008 by 1-way ANOVA; untreated versus CD3/28: *P* = 0.0043; CD3/28 versus CD3/28+6AN: *P* = 0.0008; untreated versus CD3/28+6AN: *P* = 0.177 by Tukey’s pairwise comparison). Although multiple pathways are involved in the metabolic transition from immunologic quiescence to activation, these findings suggest that PPP inhibition potently modulates the activation-induced Warburg effect in CD8^+^ T cells.

**Figure 2:**
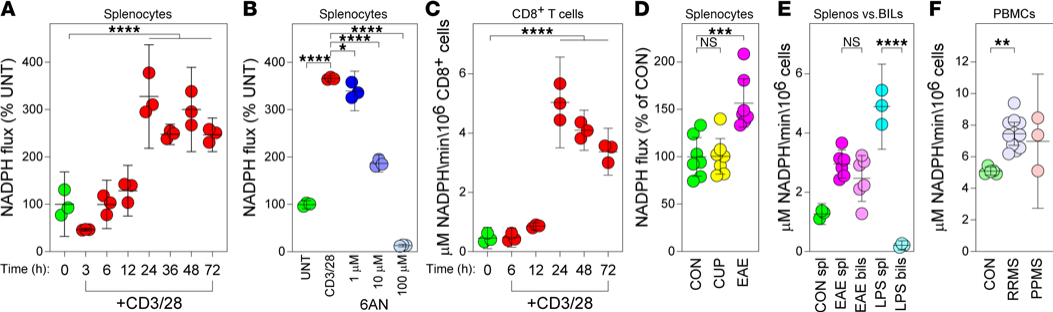
NADPH flux increases in activated T cells within the context of inflammatory demyelination. (**A**) NADPH generation (flux relative to control) determined by luciferin-based quantitative assay at 0–72 hours in CD3/28-stimulated splenocytes, normalized to flux measured at 0 hours (*n* = 3). (**B**) Relative NADPH flux at 24 hours, normalized to UNT at 24 hours, in CD3/28-activated splenocytes inhibited with 6AN (1, 10, 100 μM) (*n* = 3). (**C**) NADPH flux in splenocytes at 0–72 hours after activation (*n* = 3). (**D**) Relative NADPH flux in splenocytes obtained from untreated B6 mice (CON), animals fed cuprizone (CUP) diet for 8 weeks, or mice with MOG-EAE (EAE) at day 16 after induction; *N* = 7/condition. (**E**) Absolute NADPH flux in splenocytes (spl) or brain-infiltrating leukocytes (BILs) collected from MOG-EAE animals at day 16 after induction or at 24 hours after intraperitoneal LPS (*N* = 3 for CON spl, LPS spl, LPS BILs; *N* = 6 for EAE spl and BILs). (**F**) Absolute NADPH flux in peripheral blood mononuclear cells (PBMCs) collected from healthy individuals (CON, *N* = 5), patients with relapsing-remitting MS (RRMS, *N* = 10), or progressive patients with MS (*N* = 3). One-way ANOVA with Tukey’s pairwise comparison test was used to assess significance; *P < 0.05, **P < 0.01, ***P < 0.001, ****P < 0.0001. Data in **A**–**F** are representative of at least 3 separate experiments. Data are shown as mean ± 95% CI.

Immunometabolic adaptations downstream of CD3/28 ligation result in enhanced cofactor production to support biosynthetic and proinflammatory effector functions (27, 28), with NADPH serving as a major cofactor produced from 2 initial dehydrogenase enzymes of the oxidative branch of the PPP. NADPH is produced when glycolytic substrates are allocated to the initial, rate-limiting enzymatic reactions of the oxidative arm of the PPP (11). To understand the dynamics of PPP engagement, we assayed NADPH production in vitro in splenocyte lysates via a quantitative bioluminescent assay as a proxy for PPP engagement. We found that activation of murine splenocytes by CD3/28 ligation caused increased flux through the oxidative arm of the PPP, peaking 24 hours later but maintained through 72 hours (Figure 2A; 0 hours versus 24–72 hours, *P* < 0.0001). 6AN treatment in these cells decreased PPP flux in a dose-dependent manner when measured at the 24-hour time point (Figure 2B; CD3/28 versus 6AN 10–100 μM, *P* < 0.0001). Furthermore, we immunomagnetically enriched CD8^+^ T cells by negative selection from mixed splenocytes and found that PPP flux increased at 24 hours after activation compared with baseline (Figure 2C; 0 hours versus 24 hours, *P* < 0.0001) and then slowly decreased over 72 hours, remaining significantly elevated above baseline (Figure 2C; 0 hours versus 72 hours, *P* < 0.0001).

To interrogate PPP flux in immune cells in the context of MS-relevant mouse models, we measured PPP engagement ex vivo in splenocytes harvested at day 16 from mice with myelin oligodendrocyte glycoprotein–experimental autoimmune encephalomyelitis (MOG-EAE) and at week 8 in mice with cuprizone (CUP)-mediated demyelination (5). Splenocytes exhibited greater NADPH flux under conditions of active inflammatory demyelination (EAE) (Figure 2D; B6 versus EAE *P* = 0.0007) but not during pauci-immune demyelination (cuprizone) compared with C57BL/B6 naive controls (B6), (UNT versus CUP, *P* = 0.999). To determine if splenocytes infiltrating the CNS maintain PPP engagement, we compared PPP flux in peripheral splenocytes and brain-infiltrating leukocytes (BILs) (29). We found that BILs harvested from peak EAE maintained PPP engagement compared with peripheral splenocytes (Figure 2E; EAE splenocytes versus EAE BILs, *P* = 0.466). At 24 hours following peripheral injection of LPS to induce an acute inflammatory state, brain-infiltrating cells did not exhibit PPP engagement even though peripheral splenocytes showed evidence of strong PPP engagement (Figure 2E; LPS splenocytes versus LPS BILs, *P* < 0.0001). This suggests that infiltrating peripheral pathogenic cells in a murine CNS demyelinating disease specifically maintain an immunometabolic phenotype, though the differential composition of the cellular infiltrate in LPS (myeloid) versus EAE (lymphoid + myeloid) may contribute to the differential effect. These results prompted us to interrogate PPP engagement in human peripheral blood mononuclear cells from healthy controls (CON, *n* = 5) and individuals with relapsing-remitting MS (RRMS, *n* = 10) and primary progressive MS (PPMS). Using the quantitative PPP flux assay, we measured increased NADPH flux in RRMS compared with healthy controls (Figure 2F; CON versus RRMS, *P* = 0.0019). Furthermore, we observed a trend toward increased PPP engagement in PPMS, though these results did not reach statistical significance (CON versus PPMS, *n* = 3, *P* = 0.0556). These findings support a role for upregulation of PPP in immune cells, including both CD8^+^ and CD4^+^ T cells, in nonhuman animal models of MS and in patients with MS.

### Inhibition of the PPP blocks metabolic reorganization and suppresses effector functions in activated CD8^+^ T cells.

**Figure 3:**
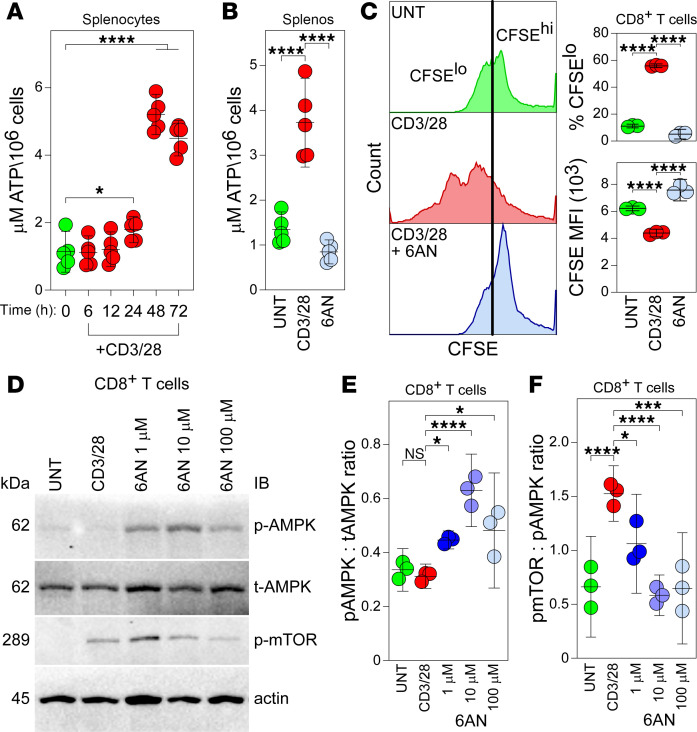
PPP inhibition blocks metabolic reorganization and suppresses effector functions in activated CD8^+^ T cells. (**A**) Timecourse of ATP generation in CD3/28-activated splenocytes (*n* = 5/condition). (**B**) ATP generation at 72 hours in unstimulated (UNT) and CD3/28-stimulated splenocytes treated with 6AN (100 μM) (*n* = 5). (**C**) Proliferation assay showing CFSE dilution at 72 hours in CD3/28-stimulated splenocytes that is blocked by PPP inhibition (*n* = 3). (**D**) Western blot analysis of unstimulated (UNT) or CD3/28-activated CD8^+^ splenocytes at 24 hours after stimulation and dose response for PPP inhibition by 6AN (1, 10, 100 μM). Blot is representative of *n* = 3/condition. Immunomagnetically enriched CD8^+^ T cells were used for this assay. (**E**) Ratio of phosphorylated AMPK (p-AMPK) to total AMPK (t-AMPK) at 24 hours under conditions in **D** (*n* = 3). (**F**) Ratio of phosphorylated MTOR (p-mTOR) to p-AMPK at 24 hours under conditions in **D** (*n* = 3). One-way ANOVA with Tukey’s pairwise comparison test was used to assess significance; *P < 0.05, ***P < 0.001, ****P < 0.0001. Data are shown as mean ± 95% CI.

Given that PPP inhibition strongly attenuated Warburg metabolism in activated T cells, we sought to characterize the effect of such inhibition on bioenergetic production and kinase activity underlying T cell proinflammatory activation. Quantitative assessment of intracellular ATP concentrations in splenocytes revealed a time-dependent increase in ATP following CD3/28 stimulation, with ATP levels elevated by 24 hours and greatly increased by 48 hours after activation (Figure 3A; UNT versus 48 hours, *P* < 0.0001). PPP inhibition at the time of activation abrogated the increase in ATP concentration, suggesting that the PPP supports TCR-driven metabolic reorganization (Figure 3B; CD3/28 versus 6AN 100 μM, *P* < 0.0001). Activated CD8^+^ T cells also proliferated in response to CD3/28 ligation (Figure 3C; %CFSE^lo^: UNT versus CD3/28, *P* < 0.0001; MFI: UNT versus CD3/28, *P* < 0.0001), and this effect was blocked by PPP inhibition (%CFSE^lo^: UNT versus CD3/28, *P* < 0.0001; MFI: CD3/28 versus 6AN 100 μM, *P* < 0.0001).

To determine if PPP inhibition altered the balance of AMPK-mTOR engagement, consistent with an effect on metabolic reprogramming, we measured AMPK phosphorylation (p-AMPK) in immunomagnetically enriched mouse CD8^+^ T cells 24 hours after CD3/28 ligation (Figure 3D). While anti-CD3/28 activation alone had no effect on the ratio of p-AMPK to total AMPK (t-AMPK) (Figure 3E; UNT versus CD3/28, *P* = 0.974), inhibition of the PPP increased this ratio in a dose-dependent manner (CD3/28 versus 6AN 1 μM, *P* = 0.044; CD3/28 versus 6AN 10 μM, *P* < 0.0001; CD3/28 versus 6AN 100 μM, *P* = 0.011). In parallel, while the ratio of phosphorylated mTOR (p-mTOR) to p-AMPK increased approximately 3-fold following CD3/28 stimulation (Figure 3F; UNT versus CD3/28, *P* = 0.0005), PPP inhibition blocked this effect in a dose-dependent manner (Figure 3F; CD3/28 versus 6AN 1 μM, *P* = 0.034; CD3/28 versus 6AN 10 μM, *P* = 0.0002; CD3/28 versus 6AN 100 μM, *P* = 0.0004).

**Figure 4:**
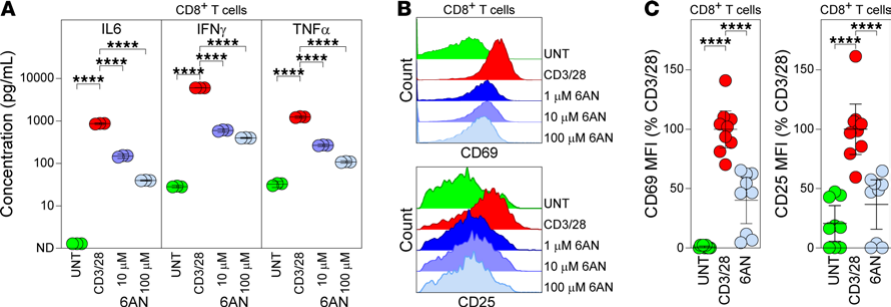
PPP inhibition suppresses effector functions in activated CD8^+^ T cells. (**A**) Cytokine levels in supernatants collected from purified CD8^+^ splenocytes at 72 hours after CD3/28 stimulation (*n* = 3). (**B**) Representative histograms showing surface expression of the activation markers CD69 (top) and CD25 (bottom) on CD8^+^ splenocytes 24 hours after CD3/28 stimulation. Top-to-bottom: unstimulated (UNT), CD3/28 stimulated, CD3/28+6AN (1 μM), CD3/28+6AN (10 μM), CD3/28+6AN (100 μM). (**C**) Surface expression of CD69 (left) and CD25 (right) on CD8^+^ splenocytes at 24 hours in unstimulated (UNT), CD3/28-stimulated, or stimulated cells treated with 6AN (100 μM) (*n* = 9). Values are normalized to percent CD3/28 of each replicate. One-way ANOVA with Tukey’s pairwise comparison test was used to assess significance; ****P < 0.0001. Data are representative of 3 separate experiments. Data are shown as mean ± 95% CI.

To assess acquisition of a T cell effector phenotype in response to CD3/28 stimulation, we measured secretion of proinflammatory cytokines after 72 hours of activation using multiplexed cytokine bead array analysis of clarified supernatants. Release of IL-6, IFN-γ, and TNF-α increased in response to CD3/28 stimulation (Figure 4A; UNT versus CD3/28, *P* < 0.0001 for all cytokines), and this effect was attenuated by PPP inhibition (CD3/28 versus 6AN 10–100 μM, *P* < 0.0001 for all cytokines). CD8^+^ T cells were also assessed at 24 hours by flow cytometry to measure cell surface expression of the acute activation markers CD69 and CD25 (Figure 4, B and C, and Supplemental Figure 2). PPP inhibition resulted in dose-dependent inhibition of surface marker upregulation (Figure 4, B and C). These results indicate that PPP inhibition impairs ATP production, blocks proliferation, and reduces cytokine secretion by activated CD8^+^ T cells, suggesting that the metabolic transition necessary to support the acquisition of effector phenotype in these cells is dependent upon the PPP.

### Inhibition of ROS generation attenuates engagement of the PPP and reduces acquisition of an effector phenotype in CD8^+^ T cells.

**Figure 5:**
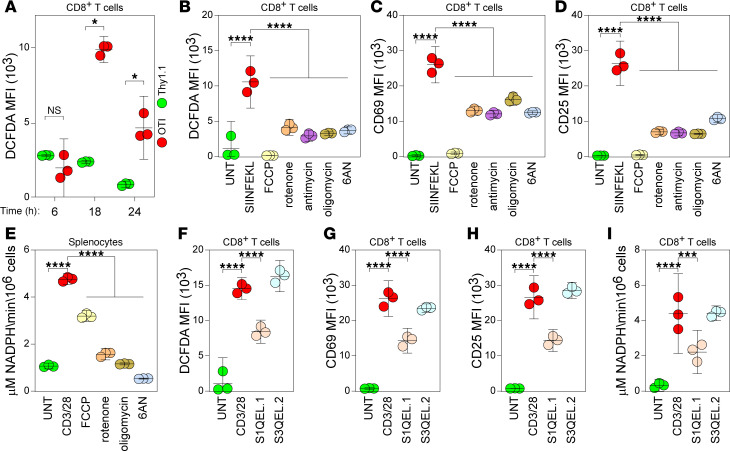
Inhibition of ROS generation attenuates PPP engagement and reduces acquisition of an effector phenotype in CD8^+^ T cells. (**A**) ROS production measured by DCFDA fluorescence in OTI CD8^+^ T cells (red) stimulated with SIINFEKL (*n* = 3). Thy1.1 nonantigen restricted CD8^+^ splenocytes (green) were used for comparison. Gating strategy is shown in Supplemental Figure 3. (**B**) ROS production at 18 hours in SIINFEKL-stimulated OTI CD8^+^ T cells treated with the mitochondrial inhibitors FCCP, rotenone, antimycin, and oligomycin or with the PPP inhibitor 6AN (*n* = 3). Unstimulated OTI T cells (green) were used for comparison. (**C** and **D**) Surface CD69 (**C**) and surface CD25 (**D**) induction at 18 hours on SIINFEKL-stimulated OTI CD8^+^ T cells treated with the same inhibitors used in **B** (*n* = 3). (**E**) NADPH production at 18 hours in CD3/28-stimulated splenocytes inhibited with FCCP, rotenone, antimycin, oligomycin, or 6AN (*n* = 3). (**F**) ROS production at 18 hours in CD3/28-stimulated splenocytes inhibited with S1QEL.1 (3 μM) or S3QEL.2 (3 μM) (*n* = 3). (**G** and **H**) Surface CD69 (**G**) and surface CD25 (**H**) induction at 18 hours on CD3/28-stimulated OTI CD8^+^ T cells treated with the same inhibitors used in **F** (*n* = 3). (**I**) NADPH production at 18 hours in CD3/28-stimulated CD8^+^ splenocytes inhibited with S1QEL.1 or S3QEL.2 (*n* = 3). One-way ANOVA with Tukey’s pairwise comparison test was used to assess significance; *P < 0.05, ***P < 0.001, ****P < 0.0001. Data in **A**–**I** are representative of at least 3 separate experiments. Data are shown as mean ± 95% CI.

In addition to the increased uptake of bioenergetic substrates such as glucose, the hyperacute phase of TCR-driven activation is associated with a burst of mitochondrial ROS production (30–32). CD8^+^ T cells isolated from OTI transgenic mice express a TCR specific for the immunodominant SIINFEKL peptide derived from OVA (33). To activate OTI T cells in an antigen-specific manner, splenocytes from OTI and Thy1.1 mice were loaded with SIINFEKL peptide, and ROS accumulation was measured by flow cytometry in CD8^+^ T cells using the redox-sensitive fluorescent indicator DCFDA (Figure 5, A and B; gating strategy shown in Supplemental Figure 3). Relative to antigen nonspecific CD8^+^ T cells derived from Thy1.1 mice, OTI antigen-specific CD8^+^ T cells exhibited increased ROS production by 18 hours after SIINFEKL stimulation (Figure 5A; OTI versus Thy1.1; *P* < 0.0001). This response was completely blocked by the mitochondrial uncoupler N-[4-(trifluoromethoxy)phenyl]carbonohydrazonoyl dicyanide (FCCP) and was attenuated by the mitochondrial complex I inhibitor rotenone, the complex III inhibitor antimycin, and by the ATP synthase inhibitor oligomycin (Figure 5B; SIINFEKL versus inhibitors, *P* < 0.0001). Notably, 6AN inhibition of the PPP also attenuated ROS production to the same levels as the mitochondrial inhibitors (Figure 5B; SIINFEKL versus 6AN 100 μM, *P* < 0.0001). We also measured the effect of mitochondrial inhibitors on the acquisition of a proinflammatory effector phenotype in antigen-stimulated OTI CD8^+^ T cells. As with ROS production, FCCP abrogated the increased CD69 (Figure 5C; SIINFEKL versus inhibitors, *P* < 0.0001) and CD25 (Figure 5D; SIINFEKL versus inhibitors, *P* < 0.0001) surface expression induced by SIINFEKL stimulation, and rotenone, antimycin, and oligomycin attenuated the expression. 6AN also attenuated the response (Figure 5, C and D). Using NADPH production as a marker of PPP engagement in splenocytes stimulated by CD3/28, we found that FCCP attenuated this pathway, while rotenone and oligomycin inhibited NADPH production nearly as effectively as 6AN (Figure 5E; CD3/28 versus inhibitors, *P* < 0.0001).

Given the observed relationship between ROS levels and PPP recruitment, we hypothesized that PPP engagement is a mechanism by which ROS production drives acquisition of an effector phenotype in CD8^+^ T cells. To test this, we blocked the specific redox sites on respiratory electron transport chain complexes I and III responsible for superoxide and H_2_O_2_ production (34, 35). Inhibition of the IQ site on complex I with S1QEL.1 attenuated ROS production in CD8^+^ T cells stimulated with CD3/28 (Figure 5F; CD3/28 versus S1QEL.1, *P* = 0.0003), but inhibition of the IIIQo site of complex III with S3QEL.2 had no effect (Figure 5F). Similarly, S1QEL.1 but not S3QEL.2 reduced the upregulation of surface CD69 (Figure 5G; CD3/28 versus S1QEL.1, *P* < 0.0001) and CD25 (Figure 5H; CD3/28 versus S1QEL.1, *P* < 0.0001) on CD3/28-stimulated OTI T cells. Finally, S1QEL.1 attenuated NADPH production in CD8^+^ T cells stimulated with CD3/28 (Figure 5I; CD3/28 versus S1QEL.1, *P* = 0.0044), suggesting that complex I–dependent generation of ROS via reverse electron transport (RET) contributes to PPP engagement upstream of the acquisition of an effector phenotype. This effect may be mediated by ROS stabilization of HIF family transcription factors (36), which are known regulators of the PPP (37).

### Inhibition of the PPP in CD8^+^ T cells prevents antigen-specific antineuronal cytotoxicity.

**Figure 6:**
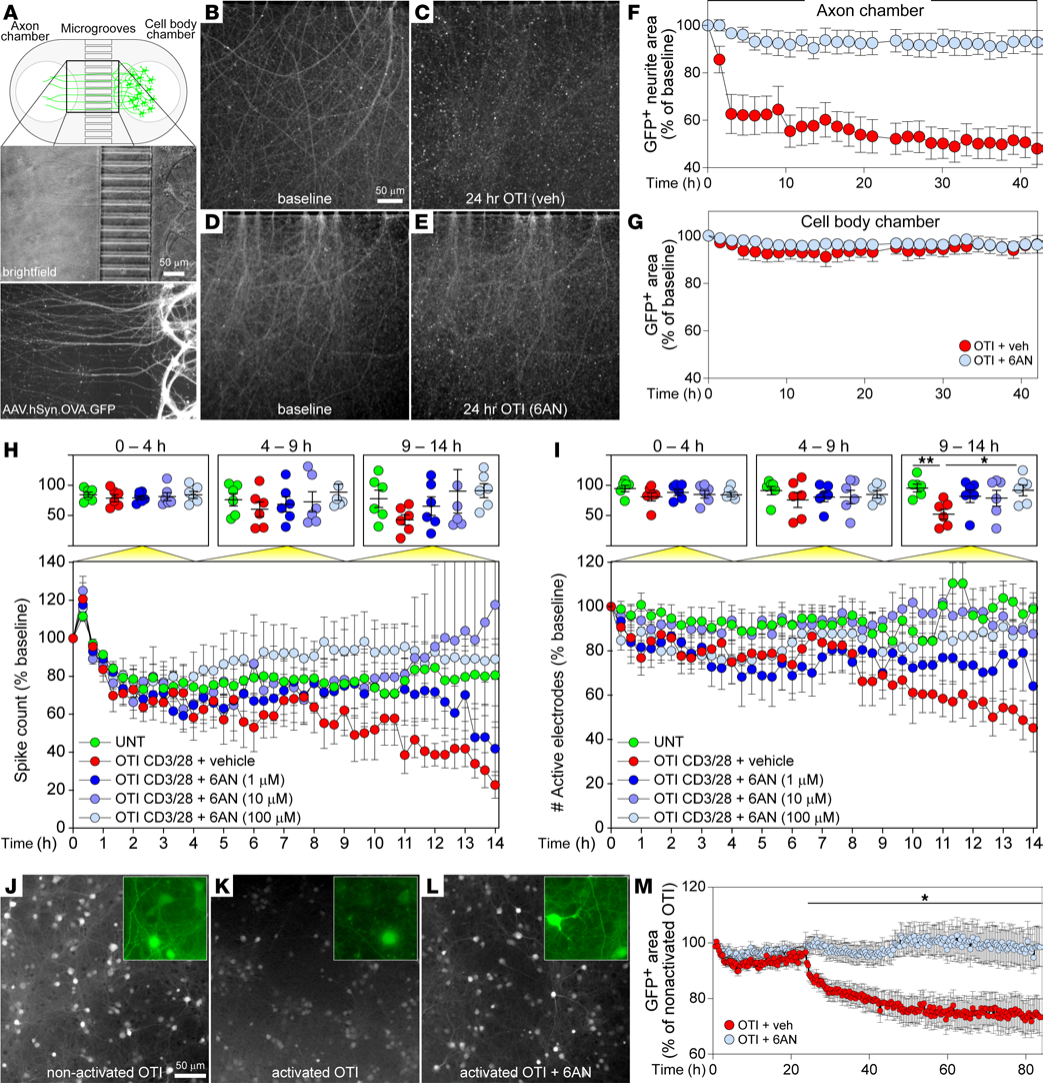
PPP inhibition in CD8^+^ T cells prevents antigen-specific antineuronal cytotoxicity. (**A**) Schematic showing neuron compartment isolated from axon compartment by microgrooves. Neurons were transduced with AAV.hSyn.OVA.GFP; CD8^+^ OTI T cells were immunomagnetically isolated prior to CD3/28 activation. (**B** and **C**) Baseline axonal GFP signal (**B**) and consequent axon injury (**C**) at 24 hours after addition of vehicle-treated activated OTI T cells (veh). (**D** and **E**) Baseline axonal GFP (**D**) and preservation of axons (**E**) at 24 hours after addition of activated OTI T cells pretreated with 6AN (100 μM). (**F** and **G**) Live cell imaging of GFP^+^ axons (**F**) and cell bodies (**G**) following addition of OTI T cells ± 6AN (100 μM) to the axon chamber (OTI+6AN, *n* = 7; OTI+vehicle, *n* = 6). (**H**) Spikes per epoch (20 minutes) in mouse cortical neurons exposed to OTI T cells (*n* = 6/condition); normalized to spike count prior to T cell addition. (**I**) Number of active electrodes normalized to activity prior to T cell addition. Top graphs in **H** and **I** show aggregated data across time tertiles (0–4 hours; 4–9 hours; 9–14 hours). (**J**–**L**) Endpoint (48 hours) image of GFP^+^ neurons following coincubation with nonactivated OTI T cells (**J**), CD3/28-activated OTI T cells (**K**), or activated OTI T cells pretreated with 6AN (100 μM) (**L**); insets show higher-magnification images. (**M**) Quantitation of neurite and neuron loss using live cell imaging of the conditions in **J**–**L**) (OTI+vehicle, *n* = 15; OTI+6AN, *n* = 15). Total GFP signal at each time point in wells incubated with nonactivated OTI T cells (**J**) was used to normalize data collected after addition of activated OTI T cells. One-way or 2-way ANOVA with Tukey’s or Dunnet’s pairwise comparison test was used to assess significance; *P < 0.05, **P < 0.01. Data are shown as 95% CI (**F**–**I**) or mean ± SEM (**M**). Scale bars are 50 μm.

We have previously shown in the cuprizone model that demyelination drives presentation of neuron-specific antigens on axonal MHC class I molecules and that antigen-specific CD8^+^ T cells infiltrate the demyelinated CNS and injure these axons (5). We have engineered a platform for testing CD8^+^ T cell–mediated axonal injury by transducing expression of chicken OVA and GFP behind the neuron-specific synapsin (Syn) promoter using an AAV vector (AAV.hSyn.OVA.GFP) (5, 33). To determine if PPP inhibition would prevent antigen-restricted CD8^+^ T cell–mediated antineuronal cytotoxicity, we seeded murine cortical neurons on 1 side of a microfluidically isolated chambering system, transduced the cells with AAV, and permitted axons to grow through microgrooves to a distal chamber (Figure 6A). After 14 days, the neurons were treated with IFN-γ for 24 hours to stimulate upregulation of axonal MHC class I loaded with SIINFEKL peptide derived from the transduced full-length OVA protein (5). Purified OTI T cells were activated ex vivo by CD3/28 ligation in the presence or absence of 6AN (100 μM) for 24 hours. Following extensive washing to remove the drug, the T cells were added to the axon chamber for 24 hours, and axon integrity was assessed at end point by microscopy (Figure 6, B–E) and by live cell imaging of GFP signal in both the axon chamber and the cell body side (Figure 6, F and G). As we have previously shown, OTI T cells do not injure neurons that are not expressing OVA (5, 33). However, within hours of coincubation with OVA transduced neurons, the activated OTI T cells reduced the total neurite area by nearly half on the axon chamber side, while the cell body side was not affected. OTI T cells pretreated with 6AN to inhibit the PPP did not injure axons (Figure 6, D–F).

To assess the effect of activated OTI T cells on neural network properties, neurons were cultured on multielectrode arrays, and the same paradigm employed above was used to drive presentation of SIINFEKL on MHC class I. Prior to addition of T cells, the basal spike count and the number of active electrodes were measured in each well, and subsequent measurements were normalized to these baseline values as an indicator of injury to the network. OTI T cells were activated by CD3/28 in the presence or absence of 6AN for 24 hours and then added to the networks at 2:1 effector-to-target ratio. The spike count and number of active electrodes remained relatively stable across conditions until the eighth hour after coincubation (Figure 6, H and I). Over the next 6 hours, the number of spikes and the number of active electrodes steadily declined in the networks incubated with activated OTI T cells. This effect was blunted or blocked in networks exposed to OTI T cells pretreated with 1 or 10 μM 6AN, and networks incubated with OTI T cells pretreated with 100 μM 6AN were indistinguishable from networks that did not see T cells (Figure 6, H and I). Binning responses across 3 epochs revealed that the effect of 6AN from the ninth to the fourteenth hour on the number of active electrodes was significant at the highest dose of 6AN relative to activated OTI T cells (Figure 6I; CD3/28 versus 6AN 100 μM, *P* = 0.0152). While other doses and the effect on spike counts trended toward an effect, the difference did not reach statistical significance due to the high variability across networks. Given the acute effect of PPP inhibition in T cells on the electrophysiological properties of the neural networks following coincubation with OTI T cells, we also measured neurite and neuronal injury in these cultures over a longer time frame (Figure 6, J–L). Neurons were transduced with AAV.hSyn.OVA.GFP, treated with IFN-γ to upregulate MHC class I loaded with SIINFEKL peptide, and then incubated with nonactivated OTI T cells (Figure 6J), CD3/28-activated OTI T cells (Figure 6K), or CD3/28-activated OTI T cells pretreated with 6AN (100 μM) (Figure 6L). PPP inhibition reduced neuronal injury induced by the T cells (Figure 6L). Live cell imaging was used to quantify this effect, revealing substantial neural preservation in the wells receiving OTI T cells pretreated with 6AN (Figure 6M). Kinetically, an early drop in GFP^+^ area in the wells receiving activated but untreated OTI cells mirrors the timing of electrophysiological disruption. This is followed by a more precipitous loss of neurons starting at around 12 hours (Figure 6M). Because GFP^+^ area can be confounded by neuronal debris, we repeated the experiment and counted the number of discrete GFP^+^ neurons. We found that, following an early increase in apparent cell counts (possibly due to cell disruption), the number of GFP^+^ neurons fell steeply starting by 5 hours after addition of simulated OTI T cells pretreated with vehicle (Supplemental Figure 4). In contrast, the addition of stimulated OTI T cells pretreated with 6AN (100 μM) did not affect neuron cell numbers, which were comparable with wells receiving unstimulated T cells or no T cells (Supplemental Figure 4). These results suggest that PPP inhibition prevents CD8^+^ T cells from acquiring the effector functions required for disruption of electrophysiological activity and loss of integrity in neurons presenting cognate antigen.

### Inhibition of the PPP in CD8^+^ T cells prevents acquisition of effector function in a mouse model of demyelinating disease.

**Figure 7:**
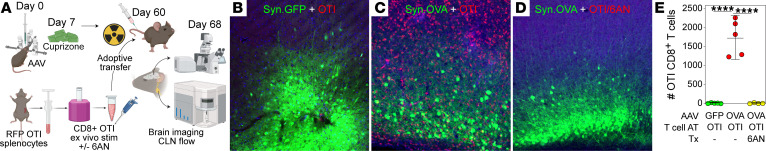
PPP inhibition prevents CNS infiltration of CD8^+^ T cells. (**A**) Schematic showing the timeline for adoptive transfer of RFP^+^CD8^+^ OTI T cells into cuprizone-demyelinated hosts transduced by intracranial injection of AAV.hSyn.OVA.GFP. (**B**) Representative image of GFP^+^ neurons and axons (green) and RFP^+^ OTI T cells (red) in the pericallosal cortex at 8 days after adoptive transfer of cells into demyelinated mice transduced with AAV.hSyn.GFP (no OVA). (**C**) Same paradigm showing a high density of RFP^+^ OTI T cells in demyelinated mice transduced with AAV.hSyn.OVA.GFP. (**D**) Preservation of GFP^+^ neurons and absence of RFP^+^ infiltrate in demyelinated mice transduced with AAV.hSyn.OVA.GFP receiving adoptive transfer of OTI T cells pretreated with 6AN (100 μM). (**E**) Number of RFP^+^ OTI T cells in the same conditions shown in **B**–**D**; *N* = 4–5 mice per condition; each symbol represents 1 mouse. One-way ANOVA with Tukey’s pairwise comparison test was used to assess significance; ****P < 0.0001. Scale bar: 100 μm (**D**).

To test the effect of PPP inhibition on neuronal injury within the setting of demyelination, we used our established adoptive transfer model (5). This model demonstrates that OTI T cells adoptively transferred into demyelinated mice traffic to the CNS and injure neurons expressing the OVA antigen — the absence of OVA expression by neurons, the absence of demyelination, or the adoptive transfer of WT CD8^+^ T cells prevents neuronal injury in this model (5). B6 Thy1.1^+^ were transduced by intracranial injection of AAV.hSyn.OVA.GFP or AAV.hSyn.GFP to drive neuron-restricted expression in the cortex (Figure 7A). Ten days later, mice were put on cuprizone diet for 7 weeks to establish robust demyelination of the corpus callosum and other CNS tracts. Mice were then irradiated to make space, and 4 hours later, they were reconstituted by tail vein injection with CD8-enriched splenocytes from Thy1.2^+^RFP^+^ OTI donors. Prior to adoptive transfer, CD8^+^ splenocytes were activated by CD3/28 ligation in the presence or absence of 6AN (100 μM). Brain, cervical lymph nodes (CLNs), and spleens were harvested 8 days later for analysis. As shown above, 6AN treatment does not adversely affect the viability of OTI T cells (Supplemental Figure 1E). We found that splenic CD8^+^ T cell numbers were not different at 8 days after transfer between mice receiving OTI or 6AN-pretreated OTI T cells (Supplemental Figure 5). Engraftment of OTI T cells (CD3^+^CD8^+^Vb5^+^Thy1.1^–^ cells) into the spleen was reduced in mice receiving 6AN-pretreated OTI T cells, but these mice still carried 5.1% ± 0.5% of splenic CD8^+^Vb5^+^ cells derived from the adoptive transfer, relative to 16.5 ± 3.5% in the mice receiving vehicle-treated OTI T cells during reconstitution (Supplemental Figure 5). This difference is consistent with the absence of stimulation-induced expansion of the 6AN-treated OTI T cells. However, despite reduced expansion to fill the lymphocyte niche, the 6AN-pretreated OTI T cells persisted in the host for at least 8 days after transfer. Within this context, adoptive transfer of CD3/28-activated OTI splenocytes into mice transduced with AAV.hSyn.OVA.GFP led to robust CNS infiltration of RFP^+^ cells (Figure 7, C–E) and apparent loss of GFP^+^ axons and neurons (Figure 7C), while adoptive transfer of activated OTI splenocytes into mice transduced with AAV.hSyn.GFP (no OVA) resulted in minimal CNS infiltration of RFP^+^ OTI T cells (Figure 7, B–E). In contrast, adoptive transfer of activated OTI splenocytes pretreated with 6AN did not result in CNS infiltration (Figure 7, D and E), and GFP^+^ cells and axons were preserved (Figure 7D).

**Figure 8:**
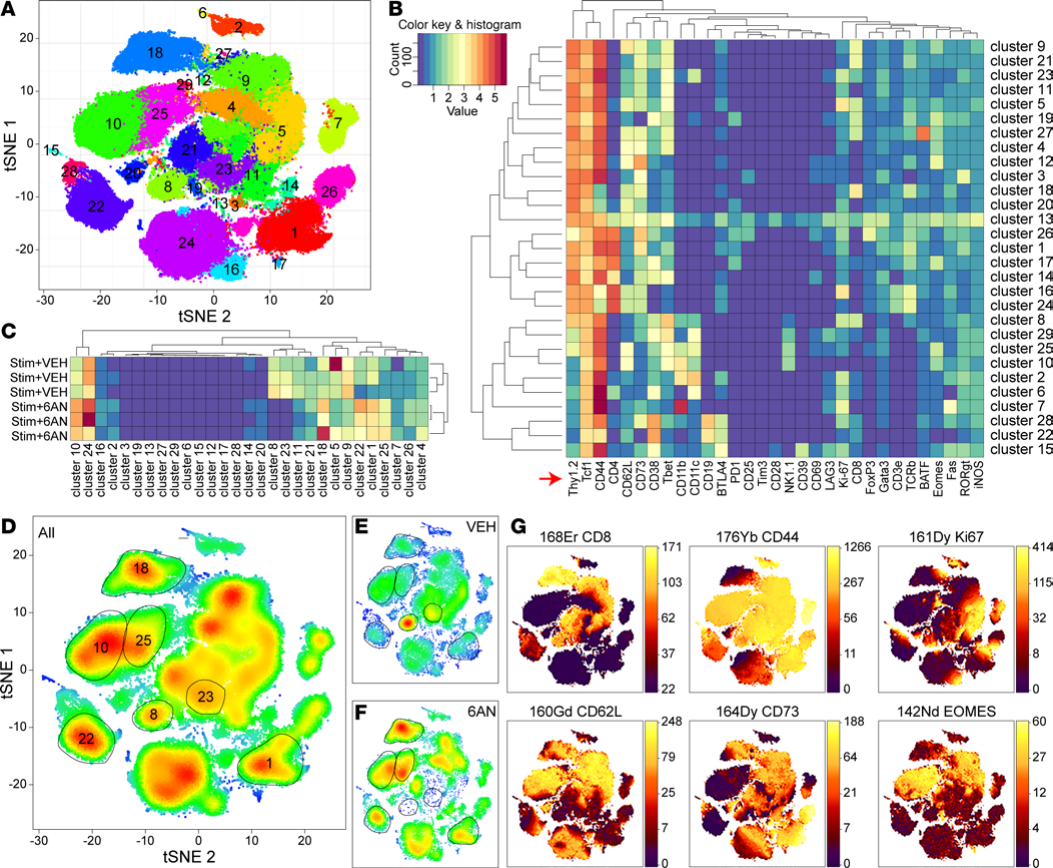
PPP inhibition prevents the acquisition of T cell effector function in vivo. (**A**) Dimensional reduction of CyTOF-based multiparametric analysis of cell surface marker expression in cervical lymph node T cells harvested from mice at 8 days after adoptive transfer of OTI splenocytes into demyelinated mice transduced with AAV.hSyn.OVA.GFP (*N* = 3 mice per condition). (**B**) Hierarchical clustering of the 16 phenotypic subsets identified in the t-SNE plot in **F**. Cells are predominantly Thy1.2^+^ (derived from the adoptive transfer donor). (**C**) Hierarchical clustering of cervical lymph node T cell subsets in mice receiving adoptive transfer of either activated OTI splenocytes or activated splenocytes pretreated with 6AN (100 μM). (**D**) Heatmap of total cellular distribution in T cells from all mice combined. (**E**) Heatmap of T cells in mice receiving adoptive transfer of vehicle-treated activated OTI splenocytes. (**F**) Heatmap of T cells in mice receiving adoptive transfer of activated OTI splenocytes pretreated with 6AN. (**G**) Localization of a subset of CyTOF surface markers aligned with the heatmaps in **D**–**F**.

The nearly complete absence of CD8^+^ T cells in the CNS infiltrate in the 6AN pretreatment condition led us to analyze the cells in the CLNs at 8 days after adoptive transfer. Cells from CLNs of mice receiving either vehicle-treated CD3/28-activated splenocytes or CD3/28-activated splenocytes pretreated with 6AN, as above, were analyzed by mass cytometry (Figure 8, A–G). Data dimensionality was reduced by t-distributed stochastic neighbor embedding (t-SNE) analysis, and 29 cell clusters were identified (Figure 8A). Hierarchical clustering of the cellular markers revealed distinct phenotypes segregated in the t-SNE clusters (Figure 8B). Hierarchical clustering also segregated the treatment conditions (Figure 8C) and, together with the t-SNE density plots (Figure 8, D–F), indicated that clusters 8 and 23 were restricted to animals receiving vehicle-treated CD3/28-activated splenocytes, while clusters 1, 10, 18, 22, and 25 were enriched in animals receiving CD3/28-activated splenocytes pretreated with 6AN. Relative to the total density of cells in each cluster (Figure 8D), there was a clear distinction between treatment groups, with clusters 8 (CD8^+^Ki67^+^Thy1.2^+^ cells) and 23 (CD8^+^Ki67^+^CD11c^+^Thy1.2^+^ cells) dominating the CLN cells in mice receiving vehicle-treated CD3/28-activated splenocytes during the adoptive transfer (Figure 8E). Further analysis of phenotypic marker expression in these clusters (Figure 8G) revealed that clusters 8 and 23 were enriched for CD8^+^CD44^+^CD62L^–^ effector memory cells. Cluster 8 was further marked by the absence of CD73 expression, and both clusters were negative for EOMES expression. While these CD8^+^ clusters were absent in the mice receiving 6AN-treated CD3/28-activated cells during the adoptive transfer, cluster 18 (CD8^+^CD62L^+^Thy1.2^+^ cells) represented abundant CD8^+^ cells that were Ki67^lo^ and expressed the lymph node retention factor CD62L (Figure 8G). Other cell types that were enriched in the lymph nodes from mice receiving 6AN-treated cells relative to mice receiving vehicle-treated cells included NK cells (clusters 10 and 25, both of which were Thy1.2^–^ or Thy1.2^lo^), CD4^+^ T cells (cluster 1), and B cells (cluster 22).

**Figure 9:**
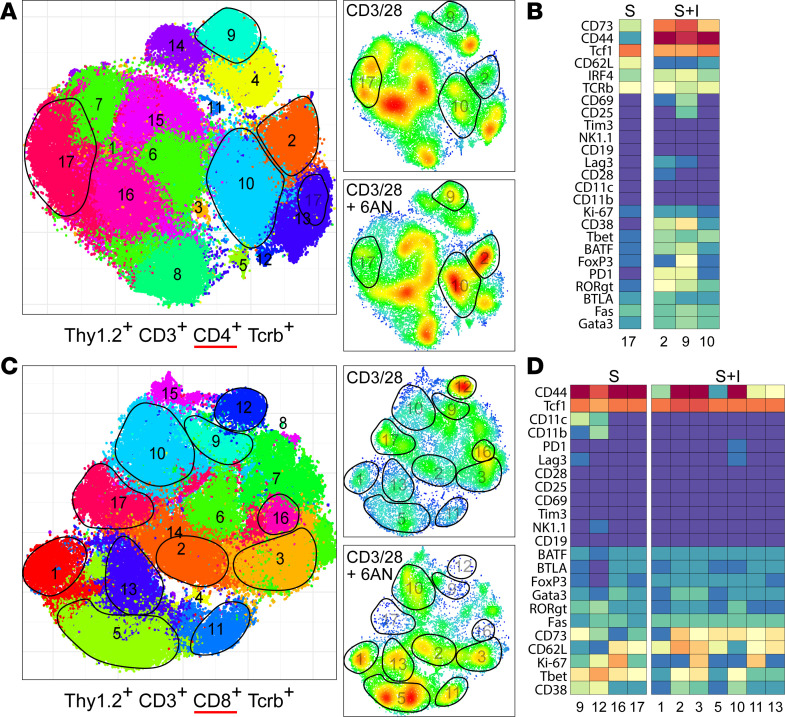
PPP inhibition prevents the acquisition of effector function in T cell subsets. (**A** and **B**) Dimensional reduction (**A**) and heatmap (**B**) of a subset of phenotypic markers on Thy1.2^+^CD3^+^CD4^+^Tcrb^+^ cervical lymph node cells from mice receiving CD3/28-activated OTI splenocytes pretreated with vehicle (*n* = 3 mice) or CD3/28-activated OTI splenocytes pretreated with 6AN (*N* = 3 mice). (**C** and **D**) Dimensional reduction (**C**) and heatmap (**D**) of a subset of phenotypic markers on Thy1.2^+^CD3^+^CD8^+^Tcrb^+^ cervical lymph node cells from mice receiving CD3/28-activated OTI splenocytes pretreated with vehicle (*n* = 3 mice) or CD3/28-activated OTI splenocytes pretreated with 6AN (*N* = 3 mice).

To further address the difference between effector cells at 8 days after transfer between mice receiving vehicle- or 6AN-pretreated splenocytes, we gated Thy1.2^+^CD3^+^Tcrb^+^CD4^+^ CLN cells (Figure 9, A and B) and Thy1.2^+^CD3^+^Tcrb^+^CD8^+^ CLN cells (Figure 9, C and D) and reassessed immunophenotypes by mass cytometry. Vehicle-pretreated CD4^+^ adoptively transferred cells largely coclustered with 6AN-pretreated CD4^+^ cells (Figure 9A), though cluster 17 cells were present in the vehicle group but absent in the 6AN group and clusters 2, 9, and 10 were found in the 6AN group but not in the vehicle group (Figure 9B). The expression of CD38 and CD69 on cluster 9 unexpectedly suggests an activation phenotype that is increased in CD4^+^ T cells in the mice receiving adoptive transfer of splenocytes pretreated with 6AN. More profound differences were observed in the Thy1.2^+^CD3^+^Tcrb^+^CD8^+^ CLN cells between the 2 conditions (Figure 9C), with nearly complete absence of clusters 9, 12, 16, and 17 in the 6AN pretreated group and the presence of clusters 1, 2, 3, 5, 10, 11, and 13 instead (Figure 9D). The most notable phenotypic difference between the groups is the elevated expression of CD73 and CD62L and the relative decrease in KI-67 on the CLN cells found at 8 days after transfer in the mice receiving 6AN-pretreated splenocytes. Further analysis revealed that CD73 was specifically reduced at 8 days after transfer on CD8^+^ CLN cells in recipients receiving vehicle-treated CD3/28-stimulated OTI splenocytes by adoptive transfer, relative to recipients receiving unstimulated cells (Supplemental Figure 6A). This effect was not observed on CD4^+^ cells (Supplemental Figure 6B). Notably, the reduction in CD73 surface expression on CD8^+^ cells was blocked by 6AN pretreatment (Supplemental Figure 6A). Moreover, the main CD73 expression effect was observed in CD45^+^CD4^–^CD8^+^ cells differentially expressing CD44, with CD3/28 stimulation inducing a large increase in CD73^–^CD44^+^ cells relative to the unstimulated condition (Supplemental Figure 6, C and D). 6AN pretreatment blocked this effect (Supplemental Figure 6, C and D). These findings are consistent with increased suppression of CD8^+^ T cell function (38), especially within the CD44^+^ central and effector memory populations. To determine if this effect was part of a more general immunosuppressive phenotype, we also assessed the surface expression of TIM2, LAG3, and PD1, but we did not observe either a stimulation- or 6AN-induced effect (Supplemental Figure 7, A and B), suggesting that the CD73 response was specifically affected by 6AN metabolic reprogramming.

### Systemic treatment with 6AN attenuates the development of functional deficits in EAE.

**Figure 10:**
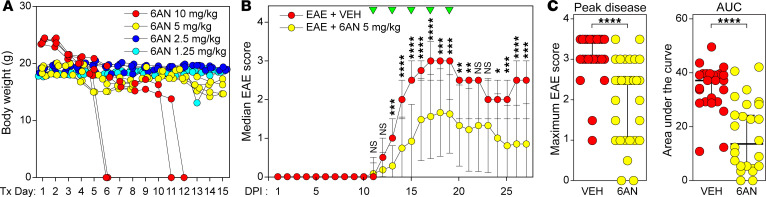
Systemic treatment with 6AN attenuates functional deficits in EAE. (**A**) Body weights of mice treated by daily i.p. injection of different 6AN doses. Red, 10 mg/kg; yellow, 5 mg/kg; blue, 2.5 mg/kg; turquoise, 1.25 mg/kg; *N* = 4 mice per condition; each symbol represents 1 animal; zero indicates animal removed from study due to euthanasia criteria or death. (**B**) Daily disability scores in mice following induction of MOG EAE. Red, EAE mice treated with vehicle (*N*, 25); yellow, EAE mice treated every other day with 6AN (5 mg/kg) (*N*, 30) starting at day 11; green triangles, treatment day; symbols show median ± 95% CI. (**C**) Peak EAE score and AUC for the data shown in **B**; vehicle-treated (red) or 6AN-treated (yellow). *N* = 25 vehicle-treated and *N* = 30 6AN-treated mice for peak disease score; *N* = 25 vehicle-treated and *N* = 25 6AN-treated mice for AUC (animals removed based on euthanasia criteria or death not included). Each symbol represents 1 animal. Two-way ANOVA with a mixed-effects model and Šidák’s pairwise comparison was used for repeated measures analysis; Mann-Whitney *U* test was used for comparison of VEH and 6AN groups; *P < 0.05, **P < 0.01, ***P < 0.001, ****P < 0.0001. Data are shown as mean ± 95% CI.

Based on the metabolic reprogramming induced by 6AN in CD8^+^ T cells and the phenotypic differences induced by 6AN pretreatment in the adoptive transfer experiment, we hypothesized that this drug would reduce or prevent functional deficits in the EAE mouse model of MS. To establish an appropriate dose, C57BL/B6 mice were treated daily for 15 days with 6AN delivered by i.p. injection at 1.25 mg/kg, 2.5 mg/kg, 5 mg/kg, or 10 mg/kg. Body weight (Figure 10A) and body condition score (not shown) were assessed daily as markers of health. Based on the observation of toxicity in the 10 mg/kg group but relatively stable maintenance of body weight in mice treated with 5 mg/kg, this dose was chosen for subsequent experiments. EAE was induced in C57BL/B6 mice using a MOG peptide (MOG_35–55_) emulsified in complete Freund’s adjuvant (CFA) and pertussis toxin treatment. Mice were treated with i.p. injection of 6AN (5 mg/kg) starting on the first day of observed symptoms (11 days postinduction [dpi]) and then every other day for 5 total doses. EAE scores were assessed every day for 4 weeks (Figure 10B). 6AN robustly suppressed the development of functional impairment in this therapeutic dosing paradigm, and the effect was maintained even after the cessation of therapy (*P* < 0.0001 for a fixed effect of treatment by restricted maximum likelihood mixed model analysis with χ^2^ = 461.2; EAE+VEH, *n* = 25; EAE+6AN, *n* = 30; data pooled from 3 separate cohorts) (Figure 10B). Mice treated with 6AN showed significantly reduced maximum disease severity (*P* < 0.0001 by Mann-Whitney *U* test; EAE+VEH, *n* = 25; EAE+6AN, *n* = 30; data pooled from 3 separate cohorts) and significantly reduced AUC (*P* < 0.0001 by Mann-Whitney *U* test; EAE+VEH, *n* = 25; EAE+6AN, *n* = 25; data pooled from 3 separate cohorts; early withdrawal animals excluded) (Figure 10C). Of the 30 total mice analyzed in the 6AN treatment group, 5 were removed from the experiment and euthanized prior to the endpoint due to poor body conditioning scores; no mice were removed from the vehicle-treated group. Analysis of brain-infiltrating cells at 36 days after induction revealed that there was no significant difference in the number of CD4^+^ or CD8^+^ cells between treatment groups (Supplemental Figure 8) (*n* = 6 per condition), though the total number of CD3^+^ cells was significantly lower in the mice treated with 6AN compared with vehicle (*P* = 0.0002 by Šidák’s pairwise comparison) and the number of CD4^+^ and CD8^+^ cells trended lower. Nonetheless, there were clearly infiltrating cells, so the absence of lymphocytic infiltrate cannot explain the protective effect of 6AN. Assessment of CD69 surface expression by flow cytometry suggests that CD8^+^ infiltrating cells were less activated in mice receiving 6AN treatment relative to vehicle-treated mice (Supplemental Figure 8).

### CD8^+^ T cells in MS patient CSF exhibit transcriptional evidence of PPP engagement.

**Figure 11:**
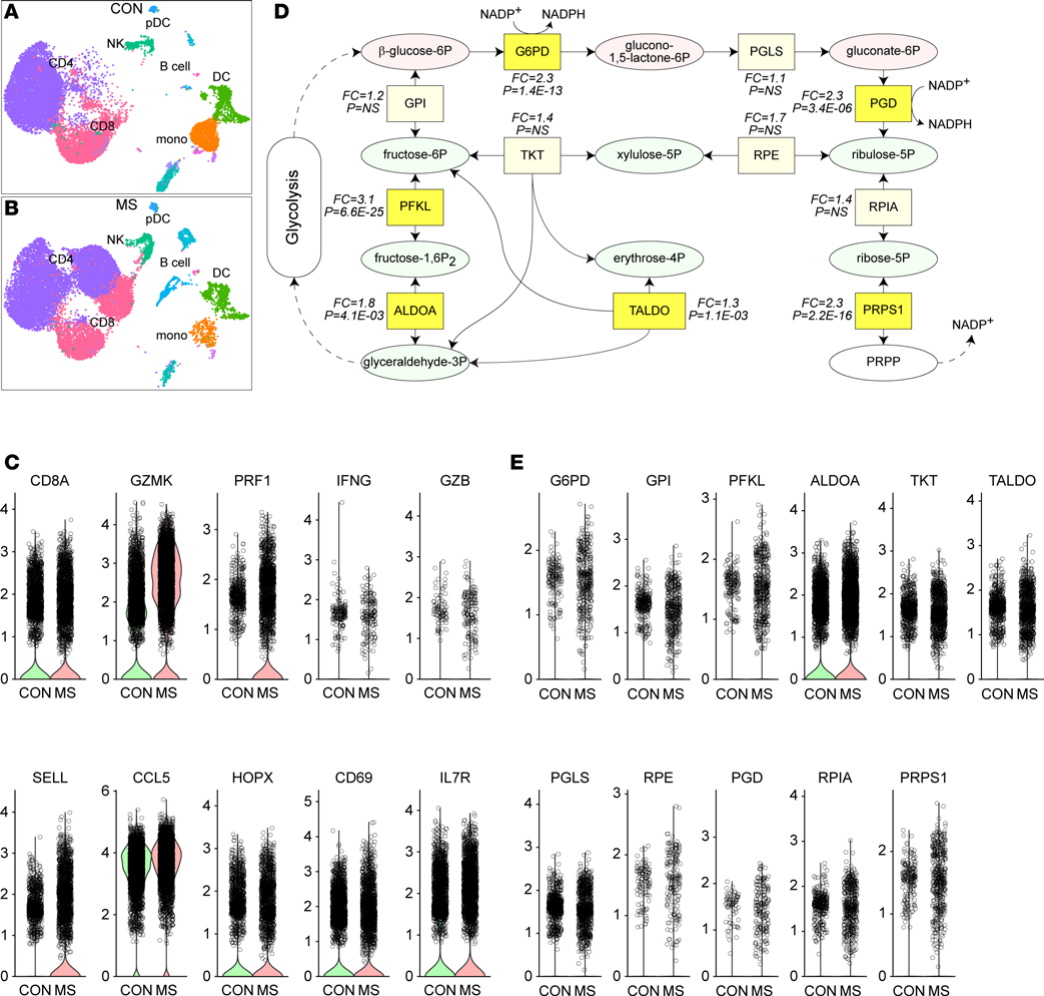
MS patient CNS-infiltrating CD8^+^ T cells exhibit PPP engagement. (**A**) Dimensional reduction (UMAP) plot showing clustering of CSF cells in controls (CON) with idiopathic intracranial hypertension (*N* = 6). (**B**) Dimensional reduction showing clusters of CSF cells from patients with MS (*N* = 6). UMAP reduction was calculated on all cells from CON and patients with MS combined (*N* = 12). CD4, CD4^+^ T cells; CD8, CD8^+^ T cells; NK, NK cells; pDC, plasmacytoid dendritic cells; DC, myeloid dendritic cells; mono, monocytes. (**C**) Relevant phenotypic marker expression in the CD8^+^ cluster from CON (green) and patients with MS (red). Gene names are shown above each plot. The *y* axis shows expression level. Each symbol represents 1 cell. To enhance clarity, all cells with zero expression have been removed from the violin plots. (**D**) Schematic showing the metabolites (ellipsoids) and enzymes (squares; marked by gene identifier) of the pentose phosphate pathway (PPP). Red metabolites are part of the oxidative arm of the PPP; green metabolites are part of the nonoxidative arm. Fold change (FC) for enzyme gene expression in MS patient CSF CD8^+^ cells relative to CON and adjusted *P* value are shown in italics. Light yellow boxes are not significant; bright yellow boxes are significant at *P* < 0.05. (**E**) Violin plots for each of the enzymes shown in **D**. Gene names are shown above each plot. The *y* axis shows expression level. Each symbol represents 1 cell. To enhance clarity, all cells with zero expression have been removed from the violin plots. Differentially expressed genes within the CD8^+^ cluster between MS and controls were identified using the Wilcoxon rank sum test via the Presto fast implementation. The average log_2_ fold change between MS and control and the Bonferroni-corrected *P* value for the comparison were calculated.

Given our observations regarding the influence of PPP inhibition on CD8^+^ T cell infiltration and disease progression in 2 mouse models of demyelination, we hypothesized that CD8^+^ T cells within the CNS of patients with MS would likewise exhibit evidence of PPP engagement. To test this hypothesis, we used a previously published dataset (39) assessing the single-cell transcriptional profile of cells isolated from CSF collected from patients with MS (*n* = 6) or patients with idiopathic intracranial hypertension (IIH) (*n* = 6) (Supplemental Table 2). Unsupervised dimensional reduction, clustering, and cell type classification on 35,332 cells integrated across all 12 patients identified distinct populations of immune cells in the CSF. While cell types were broadly comparable between the patient groups, clearly unique populations of CD8^+^ and CD4^+^ T cells were present in MS patient CSF that were absent in IIH CSF (Figure 11, A and B). Of the 19,312 cells analyzed from patients with MS, 4,107 were clustered as CD8^+^ T cells (21%); of 16,020 cells analyzed in IIH controls, 3,798 were in the CD8^+^ T cell clusters (24%). Further analysis of phenotypic markers in the CD8^+^ subset revealed increased GZMK (2.1-fold MS versus IIH, *P* = 5.5 × 10^–209^), PRF1 (3.1-fold, *P* = 6.9 × 10^–105^), SELL (2.6-fold, *P* = 7.1 × 10^–67^), CCL5 (1.2-fold, *P* = 2.3 × 10^–21^), and IL7R (1.3-fold, *P* = 1.7 × 10^–6^) and decreased CD69 (0.8-fold, *P* = 1.4 × 10^–33^) in the patients with MS relative to those with IIH (Figure 11C). IFNG, GZB, and HOPX levels were not significantly different between the patient groups.

To determine if MS patient CNS-infiltrating CD8^+^ T cells exhibit a transcriptional signature consistent with PPP engagement, we identified 11 key enzymes involved in the oxidative and nonoxidative arms of the PPP (Figure 11D). Two key enzymes involved in the oxidative arm of the PPP and the production of NADPH were significantly upregulated (G6PD: 2.3-fold MS versus IIH, *P* = 1.4 × 10^–13^; PGD: 2.3-fold, *P* = 3.4 × 10^–6^) (Figure 11E). In addition, TALDO and PRPS1 were also upregulated in MS CNS-infiltrating CD8^+^ T cells, suggesting a systematic skew toward PPP engagement in these cells. Furthermore, HIF1A was upregulated (3.2-fold, *P* = 4.5 × 10^–19^) in MS patient CNS-infiltrating CD8^+^ T cells relative to controls, suggesting that this factor may act as an upstream regulator of the oxidative and nonoxidative arms of the PPP in response to RET-ROS signaling, as others have suggested (36, 37).

## Discussion

Despite the availability of numerous disease-modifying therapies approved for the treatment of patients with MS, stopping progressive and irreversible loss of neurologic function remains a critical unmet need for these patients. Histopathological data and findings from nonhuman animal models implicate CD8^+^ cytotoxic T cells in the pathogenesis of axonal injury and neuronal damage underlying irreversible clinical progression associated with relapse in people with MS (4, 5, 40–44). Moreover, evidence also indicates that CD8^+^ T cells persist in the CNS even in lesions that are not actively demyelinating, as well as in normal-appearing white and gray matter (5), suggesting that these cells may contribute to progression independent of relapse activity. CD8^+^ T cells are also implicated in the pathogenesis of autoimmune neurological diseases associated with intracellular antigens (45, 46), suggesting broader relevance to immunopathogenesis within the CNS. In parallel, our understanding of the dynamic role of immunometabolism in controlling T cell proliferation and effector function (12) has led to the development of metabolic reprogramming strategies for cancer (47) and autoimmune diseases (48). Much of the focus for immunomodulation and metabolic reprogramming in autoimmunity has been on CD4^+^ T cells (49). In this study, we found that CD8^+^ T cells upregulated anabolic glycolysis and engaged the PPP upon activation by CD3/28 ligation, and pharmacologic inhibition of the PPP with 6AN suppressed glycolytic capacity and reduced glucose uptake in activated T cells. Activation increased flux through the oxidative arm of the PPP in CD8^+^ T cells, as measured by NADPH production and stable isotope tracing. PPP inhibition with 6AN prevented activation-induced metabolic reprogramming, attenuated ROS production, reduced ATP production, blocked proliferation, reduced inflammatory cytokine secretion, and prevented acquisition of an activation phenotype. Inhibition of the PPP also prevented antigen-specific CD8^+^ T cell–mediated neuronal injury, both in vitro and in a mouse model of demyelination, and antigen-specific CD8^+^ T cells treated ex vivo with 6AN largely failed to infiltrate the CNS following adoptive transfer in this model, though they survived after transfer and expanded within the CLNs and spleen (Figures 8 and 9, and Supplemental Figure 5). Failure of the transferred cells to accumulate in the demyelinated CNS even after in vivo wash out of 6AN drug effects suggests that acutely frustrating initial antigen stimulation by impairing PPP flux may drive the T cells into a nonresponsive or anergic fate. Future work will need to refine this hypothesis, but our initial observation that CD73 expression on CD8^+^ T cells is altered by PPP inhibition (Supplemental Figure 6) without concomitant alteration of TIM3, LAG3, or PD1 expression (Supplemental Figure 7) suggests that 6AN does not simply induce an exhausted phenotype. In parallel, systemic treatment with 6AN attenuated the development of functional deficits in the MOG-EAE model of demyelination, and while overall numbers of CNS infiltrating CD3^+^ T cells were reduced in 6AN-treated mice (Supplemental Figure 8), both CD4^+^ and CD8^+^ T cells were still present within the CNS, suggesting that global suppression of T cell infiltration is not the underlying mechanism of action for the PPP inhibition effect. Finally, single-cell transcriptional profiling of CD8^+^ T cells in CSF from patients with MS indicated a skew toward PPP engagement. Overall, these findings implicate the PPP as a critical immunometabolic rheostat that controls CD8^+^ T cell–mediated injury within the demyelinated CNS.

Antigen-driven T cell activation recruits promitotic kinase activity, resulting in the acute upregulation of progrowth signaling pathways that include MAPK, HIF1α, MYC, and the PI3K/AKT/MTOR axis (15). Our kinetic analysis of PPP engagement using a quantitative bioluminescence assay (50, 51) revealed peak pathway engagement by 24 hours that was maintained through 72 hours, consistent with the kinetics of CD28-, AKT-, and HIF1α-mediated reinforcement of aerobic glycolytic metabolism (16). Following TCR ligation, the balance of downstream master regulators AMPK and MTOR is biased toward mTOR predominance in T cells, a shift known to support T cell effector responses, differentiation, and memory formation (52, 53). We demonstrated that inhibition of the PPP induces phospho-AMPK and inhibits CD3/28-driven MTOR upregulation, similar to the inhibition of ECAR that occurs following treatment with AICAR and metformin. Since MTOR activity is directly inhibited by AMPK, the upregulation of AMPK is hypothesized to limit inflammation through a mechanism of pseudostarvation analogous to conditions of glucose depletion (54). Given that AMPK activity is regulated by the concentration of ATP, the effect of PPP inhibition on mTOR upregulation may be secondary to ATP depletion in the setting of increased metabolic demand. Consistent with this hypothesis, inhibition of the PPP also decreased activation-associated ATP generation, though clarification is needed to determine if this is a primary effect or secondary to other PPP-mediated functions.

Within the immune system, ROS generation is required for acquisition of adaptive immune cell effector functions (55, 56). Mitochondria are considered a primary source of ROS (30–32), with up to 2% of oxygen respiration yielding such molecules due to nonproductive electron leakage (57, 58). Although generated in mitochondria, ROS passively diffuse across subcellular compartments (H_2_O_2_) or diffuse via voltage-dependent anion channels (superoxide), linking mitochondrial activity to the oxidative tone of the cytosol (59). Along the electron transport chain, respiratory complexes I and III are key sites for the production of ROS involved in the acquisition of distinct proinflammatory phenotypes (60). For example, ROS generated at complex I stabilize HIF1α and increase IL-1β production in LPS-treated macrophages, while complex III–derived reactive oxygen species drive IL-2 production in T cells secondary to NFAT nuclear translocation (61). HIF1α also regulates the oxidative and nonoxidative arms of the PPP (37). We show that inhibition of complex I with rotenone, complex III with antimycin A, or dissipation of the proton motive force with FCCP all contributed to decreased CD3/28 ligation–induced ROS generation and PPP engagement. Notably, however, directly inhibiting the PPP with 6AN appeared to more strongly attenuate acquisition of an activated T cell phenotype compared with any individual mitoROS inhibitor, suggesting that the PPP may serve as a central integrator of oxidative tone from multiple subcellular compartments during the acquisition of proinflammatory effector functions in CD8^+^ T cells.

Among mechanisms of mitochondrial ROS generation, RET represents a conditional mode that results in ROS production at complex I and III. Although this mode of generation operates within the physiological context of hypoxia sensing (62) and in the pathological setting of reperfusion injury (63), no investigation to date has explored the relevance of RET in CD8^+^ T cells within the context of CNS autoaggressive disease. The complex I inhibitor rotenone targets both the I_F_ and I_Q_ sites and, therefore, cannot be used to clarify the specific role of forward versus reverse modes of electron transport (57, 64). To address this, we used S1QEL.1 to specifically inhibit the IQ site on complex I and S3QEL.2 to inhibit the IIIQo site on complex III (34, 35). Although inhibition of ROS production in response to CD3/28 ligation was not achieved by blocking RET at complex III, S1QEL.1-mediated inhibition at complex I attenuated both ROS production and acquisition of an activated phenotype in CD8^+^ T cells. Furthermore, inhibition of RET at complex I also blocked PPP engagement. These findings indicate that CD8^+^ T cell activation involves a RET/ROS/PPP axis that may have relevance for therapeutically targeting autoaggressive T cell–mediated diseases. For example, electron donors distal to complex II, including the teriflunomide target DHODH, also contribute to RET, suggesting that the efficacy of this therapy in MS (65) may be at least in part associated with modulation of the RET/ROS/PPP axis. Perhaps more importantly, targeting RET to affect T cell immunometabolism while preserving forward electron transport may confer neuroprotection in autoaggressive T cell–mediated diseases without compromising neuronal bioenergetic integrity (66, 67).

Our observation of increased PPP engagement in circulating CD8^+^ T cells isolated from patients with MS, coupled with evidence of a transcriptional program that is consistent with PPP activation in CD8^+^ T cells isolated from the CSF of patients with MS (Figures 2 and 11), suggests that this pathway is pathogenically relevant. Additionally, our findings suggest that CD8^+^ T cells may be more reliant on PPP metabolic reprogramming and Warburg metabolism for activation and effector function than CD8^–^ immune cell populations (Figure 9). However, it is important to recognize that 6AN treatment also likely disrupts PPP engagement in other cell types that have relevance to MS. For example, the reduced EAE clinical scores observed in 6AN-treated mice may be partially explained by impairment of PPP engagement in CD4^+^ T cells changing the initial slope of disease onset (Figure 10). While the function of CD8^+^ T cells remains somewhat unclear in EAE, with some evidence for a pathogenic role and some evidence for a regulatory role, given that 6AN treatment suppressed disease severity rather than exacerbating it, as would be expected if treatment reduced Treg function, we conclude that PPP inhibition may be a unique strategy for targeted inhibition of neuropathogenic CD8^+^ T cells. Recognizing the caveats associated with drug testing in all nonhuman animal models of MS, it is still notable that we observed neuroprotection following pharmacological inhibition of the PPP in 2 very distinct demyelinating disease models. Indeed, the primary limiting factor for using 6AN in mice with EAE was iatrogenic toxicity; treated mice showed remarkable protection of neurologic function right up until the point where they acutely died or required euthanasia. While we are not suggesting the use of 6AN as a therapy in patients with MS, PPP inhibition via this drug has been tested in various cancers for many years, with evidence of a “safe” dosing regimen that could be tolerated daily without side effects for up to 4 weeks reported in 1961 (68). While drugs such as 6AN or polydatin may carry unacceptable risks of toxicity, our findings support further investigation into the development of strategies for modulating the PPP in autoaggressive CD8^+^ T cells to protect the CNS. In addition to potentially taming the T cell–mediated neural injury that underlies progression in MS (5), pharmacological inhibition of the PPP may be a powerful direct approach or adjunct therapy in patients with rapidly progressive immune-mediated neurodegenerative diseases mediated by autoimmune CD8^+^ T cells (69), including anti-GAD encephalitis and the paraneoplastic encephalitides involving autoimmune responses against targets such as KLHL11 (70), Hu, Yo, and Ma/Ta (71, 72).

## Methods

Supplemental Methods are available online with this article.

### Sex as a biological variable.

Sex was not analyzed as a variable in this study. Female mice were used for EAE based on literature indicating more robust demyelination, larger immune cell infiltration, and more reproducible disease severity in females compared with males (73).

### Statistics.

All graphs show mean ± 95% CI unless otherwise stated. *P* values less than 0.05 were considered significant. Biological (*N*) and technical (*n*) replicates are stated in the figures. Unless otherwise noted, data from in vitro experiments are representative of at least 3 independent experiments. Statistical analyses were performed using GraphPad Prism v9. Normality was determined using the Shapiro-Wilk test, and normally distributed data were checked for equal variance. Parametric tests were applied to data that were normally distributed and of equal variance. Multiple independent groups were compared using 1-way or 2-way ANOVA with Tukey’s multiple-comparison test, and 2-group comparisons were made using 2-tailed unpaired Student’s *t* test or Welch’s student *t* test. Specific tests used are detailed in figure legends. Electrophysiologic data and EAE time course data were analyzed using 2-way ANOVA with a mixed-effects model for repeated measures analysis. Correction for multiple comparisons was performed by Dunnett’s method or Šidák’s multiple comparison test. For single-cell RNA-Seq analysis, differentially expressed genes were identified using the Wilcoxon rank sum test, and the Bonferroni-corrected *P* value for the comparison was calculated and filtered on *P* < 0.05.

### Study approval.

All human samples were obtained from individuals providing written informed consent following protocols approved by the Mayo Clinic IRB. All animal experiments were approved by and performed in accordance with the Mayo Clinic IACUC and the NIH guidelines.

### Data availability.

All data are available in the main text or the supplemental material. Individual data points for each figure are available in the Supporting Data Values file, and uncropped Western blot images are available as supplemental material. Please contact the author for any further material or data requests.

## Author contributions

EMG, BDSC, and CLH conceptualized the study. EMG, BDSC, and CLH developed the methodology, carried out the investigation, performed initial analysis of the data, and generated initial figure drafts. SP, MSW, CMP, and SAM performed experiments and initial analysis of data. CLH validated the analyses and generated the final figures. CLH acquired funding, provided project administration, and supervised the project. EMG, BDSC, and CLH wrote and edited the manuscript. All authors approved the manuscript. The order of co–first authors was determined by the relative role in conception and design of the original study, with EMG being the study initiator.

## Supplementary Material

Supplemental data

Unedited blot and gel images

Supporting data values

## Version 1 06/10/2025

In-Press Preview

## Version 2 07/22/2025

Electronic publication
