# Supplementary material for: Pentose phosphate pathway inhibition metabolically reprograms CD8+ T cells and disrupts CNS autoimmunity
Source: JCI Insight. 2025 Jun 10;10(14):e184240. doi: 10.1172/jci.insight.184240 (PMC12288970; doi:10.1172/jci.insight.184240)
Supplement: Supplemental data [file jciinsight-10-184240-s149.pdf]

Supplemental Methods

Human peripheral blood mononuclear cells (PBMCs)

PBMCs were isolated as described (1). Cryorecovered cells were rested at 37°C for 3 hours, then resuspended in 3 mL warm lymphocyte medium (RPMI-1640 (Invitrogen 11875-093), 3% heat-inactivated FBS (Life Tech/Gibco, #10437-028), 1% sodium pyruvate (Sigma, #S8636), 1% non-essential amino acids (NEAA) (Sigma, #M7145-100ml), 100 U/mL penicillin + 100 ug/mL streptomycin (Gibco, #15140-122), and 25 µM β-mercaptoethanol (BME; Gibco, #21985023)) containing 10 ng/mL human IL-7 (Peprotech 200-07). Cells were incubated for 72 hours before subsequent analysis.

Mice and demyelination models

C57BL/6 wild-type (WT; B6; Stock No. 000664), B6.PL-Thy1/CyJ (Thy1.1; Stock No. 000406), and C57BL/6-Tg(TcraTcrb)1100Mjb/J (OTI, stock # 003831) mice were obtained from the Jackson Laboratories (Bar Harbor, ME). Mice were fed cuprizone diet starting at 10 weeks of age, as described (2). Experimental autoimmune encephalomyelitis (EAE) was induced in female B6 mice at 11-13 weeks of age, as described (2). Mice used for splenocytes ranged from 8-20 weeks of age. All mice were group housed under controlled temperature and humidity with a 12-h light/dark cycle. Mice were provided ad libitum access to food and water.

Intracerebral AAV injection

Anesthetized adult mice (>10 weeks of age) were injected with 1.5 µL PBS containing $3x10^9$ gene copies of AAV1.Syn.OVA.GFP (2) at a rate of 0.5 µL/min using a 600 series 5 µL Hamilton syringe (No. 7633-01) and a Quintessential Stereotactic Injector (QSI) pump (cat #53311). Virus was delivered 1.0 mm left of Bregma at a depth of 1.0 mm below the surface of the brain. The needle was held in place for 5 min following injection to prevent reflux upon needle removal.

Pharmacological preparation

Chemicals were reconstituted as follows: 5-Aminoimidazole-4-carboxamide 1-B-D ribofuranoside (AICAR) (Sigma, #A9978) in PBS, metformin HCl (MET) (Sigma PHR1084) in PBS, 6-aminonicotinamide (6AN) (Cayman Chemical, #329-89-5) in DMSO, polydatin (polyD) (Cayman Chemical, #13932) in DMSO, oligomycin A (Sigma, #75371) in ethanol, N-[4-(trifluoromethoxy)phenyl]carbonohydrazonoyl dicyanide (FCCP) (Sigma, #C2920) in DMSO, rotenone (Sigma, #R8875) in DMSO, antimycin A (Sigma, #A8674) in DMSO, S1QEL.1 (Cayman Chemical, #20982) in DMSO, and S3QEL.2 (Cayman Chemical, 18556) in DMSO.

Murine immune cell isolation and activation

Brain-infiltrating leukocytes were isolated as described (3). Spleens and cervical lymph nodes were dissociated by Dounce homogenization in RPMI base media (Gibco, #11875085) containing 15% heat-inactivated FBS, 5% sodium pyruvate, 5% NEAA, 5% penicillin/streptomycin, and 25 µM BME. Following red blood cell lysis, cells were passed through a 40 µm cell strainer (Falcon; #352340) to obtain a single cell suspension. Cells were plated at 1-3 x 10^6 cells/well in 48-well tissue culture plates or 0.5-1 x 10^6 cells/well in 96-well plates. When applicable, $CD8^+$ T cells were magnetically enriched using the EasySep Mouse $CD8^+$ T cell Isolation Kit (Stem Cell Technologies, #19853). For T cell activation, wells were coated with anti-CD3 (BD Biosciences, #145-2C11) and anti-CD28 (Biolegend, #102116) antibodies at 10 µg/ml in PBS. For peptide stimulation of OTI $CD8^+$ T cells, splenocytes were stimulated with 1 ng/mL SIINFEKL peptide (Anaspec, AS-60193-1) and 10 ng/mL IL-2 (Prepotech, #212-12).

T cell transfers

For adoptive transfer, immunomagnetically enriched OTI (Thy1.2) or OTI RFP (mt/mG) $CD8^+$ splenocytes were activated by anti-CD3/28, washed, counted, spun at 400g for 4 min, and resuspended in 50% FBS/ PBS at 10^7 cells/mL. Recipient Thy1.1 or B6 mice were irradiated with 400 rads 4 hours prior to intraperitoneal injection of 300 µL containing 3 x 10^6 $CD8^+$ T cells. Counts and viability were confirmed by flow cytometry.

Extracellular flux assay

The extracellular acidification rate (ECAR) was measured using a Seahorse XFe bioanalyzer (XFe96, Agilent Technologies). 1 $x10^6$ splenocytes/well in Seahorse RPMI media (Agilent, #103576-100) were seeded onto Cell-Tak (Corning, #354240)-coated Seahorse 96 well plates and pre-incubated at 37°C for 60 min in the absence of CO_2. XF media was supplemented to achieve a final concentration 1 mM glutamine (ThermoFisher, #25030149) and 1 mM sodium pyruvate (Sigma, #S8636). ECAR was measured 24 hr after in vitro activation

with 10 µg/mL plate-bound anti-CD3/28. ECAR measurements were obtained under basal conditions and after the sequential addition of 10 mM glucose, 2 µM oligomycin (Sigma #75351), and 20 mM 2-DG (Cayman #14325). ECAR measurements were normalized to µg protein/well. Glycolytic capacity was calculated as the difference between the maximum ECAR following addition of oligomycin and the minimal ECAR prior to addition of glucose.

NADPH bioluminescence assay

NADPH flux assay was performed as described (4, 5), with modifications. 10^6 splenocytes were plated in 96-well black, clear-bottom plates and 50 µL detection reagent lacking the NADP cyclin enzyme and NADP cycling substrate was added (Promega, #G9082). G6P (40 µM) (Sigma, #G7879) and NADP+ sodium salt hydrate (20 µM) (Cayman Chemical, #10004675) in 100 mM Tris pH 8.0 was added to each well. Luminescence was measured using a SpectraMax M3 spectrophotometer (Molecular Devices) with 500 ms integration at 37°C and results were analyzed during the linear phase of the kinetic assay. Concentration of NADPH was determined from a standard curve generated using NADPH sodium salt (Cayman Chemical, #9000743).

Adenosine triphosphate (ATP) detection assay

ATP was measured using the CellTiter-Glo 2.0 assay kit (Promega# G9241). Briefly, 10^6 splenocytes/well were plated in black clear bottom 96-well plates 24 hr after anti-CD3/28 activation. An equivalent volume of CellTiter-Glo 2.0 reagent was added to each well without washing off media. Reactions were incubated at room temperature for 10 min on a shaker. Luminescence was measured using a SpectraMax M3 spectrophotometer with 500 ms integration.

Stable isotope tracing

T cells isolated from C57BL/6 spleens were resuspended in complete lymphocyte media and 5×10^7 cells were plated in a 75 cm^2 cell culture-treated flask that was pre-coated overnight with mouse anti-CD3 (purified anti-mouse CD3, Biolegend #100202) and anti-CD28 (Purified anti-mouse CD28 Antibody, Biolegend #102102) antibodies at a concentration of 10 mg/mL each. Unstimulated cells were plated in uncoated flasks under the same conditions. Cells were treated with 6-aminonicotinamide (6AN; 100 µM) (Cayman #329-89-5) at the time of plating. Three replicates were used for each experimental condition. On day 1 after plating the cells were washed with glucose-free RPMI (ThermoFisher Scientific, #11879020) and transferred to new flasks containing glucose-free RPMI, dialyzed low-glucose FBS (assayed at 11 µg/mL glucose) (ThermoFisher Scientific, #A3382001), and D-glucose-1,2-$^{13}C_2$ (2 mg/mL) (Millipore Sigma, #453188). The cells were incubated in this media at 37°C for 4 hours with fresh 6-aminonicotinamide. Cells were washed once in cold PBS and then snap frozen. Samples were kept at -80°C until subsequent analysis. To measure pentose phosphate pathway metabolites, we employed a previously published liquid chromatography – mass spectrometry method, with minor modifications (6). In brief, cells were lysed in PBS and proteins were precipitated by adding chilled methanol-acetonitrile. Supernatants were clarified by high-speed centrifugation and then dried on a speed vacuum concentrator. Following reconstitution in running buffer, samples were separated on an Agilent Zorbax Extend C18 column (2.1 mm x 150 mm, 2 µm), followed by metabolite detection on an Agilent 6460 triple quadrupole mass spectrometer. Data were collected in negative electrospray ionization mode using dynamic multiple reaction monitoring. Mole percent enrichment was calculated based on peak area for each analyte relative to the corresponding isotope-labeled peak. Residual samples from the PPP analysis were dried and then sequentially derivatized with ethoxyamine and N-Methyl-N-(t-butyldimethylsilyl)trifluoroacetamide + 1% t-butyldimethylchlorosilane. Samples were then analyzed on an Agilent 5977B GC/MS using electron impact ionization in single ion monitoring mode. Lactic acid isotopomers were quantified as previously described (7), based on peak areas of lactic acid isotopologues (8).

Flow cytometry, cytometric bead assay, and CyTOF

Splenocytes were blocked with 1% BSA and 1 µg 2.4G2 antibody (CD16/CD32 BD Pharmingen, #553141). Cells were labeled with antibodies diluted 1:100 in block. The following antibodies were used (all BD Biosciences): FITC anti-CD69 (#557396), PE anti-CD25 (#558642), PerCP anti-CD8a (#551162), APC anti-CD8a (#553035), APC anti-Thy1.1/CD90.1 (#561409). For most experiments, viability was assessed with propidium iodide (Invitrogen, #P3566) or Fixable Viability Stain 700 (BD Biosciences, #564997) diluted 1:1000 in PBS. For analysis of T cell survival following 6AN treatment, cell death was measured using AnnexinV-FITC (BD Biosciences #556419) in AnnexinV binding buffer (BD Biosciences #556454) and propidium iodide (Miltenyi Biotech #130-093-233). Some samples were labeled with 5 µM 2',7'– dichlorofluorescein diacetate

(DCFDA) (ThermoFisher, #D399) in PBS, 100 mM 2-NBDG in PBS (Cayman Chemical, #11046), or 5 μM carboxyfluorescein succinimidyl ester (CFSE) (BD Biosciences, #565082). Samples were run on an Attune NxT Acoustic Focusing flow cytometer (Thermo Scientific) running Attune NxT Software (v2.5). All samples were analyzed using FlowJo v10 (FlowJo LLC, Ashland, OR). Cytometric bead array analysis of proinflammatory cytokines was performed on an Accuri C6 flow cytometer (BD) using the Mouse Inflammation CBA kit (BD Biosciences, #552364). Cytometry by time-of-flight analysis was performed as described (2).

Western blot assay
10^6 splenocytes were washed with PBS and lysed in lysis buffer (Life Technologies, FNN0021) containing 1 mM phenylmethylsulfonyl fluoride (PMSF) (Fisher, NC9953637), 1 mM sodium orthovanadate (Sigma, #S6508), 1 mM sodium fluoride (Sigma, #S7920), 10 μg/mL aprotinin (Roche, #10336624001), and 1 μg/mL leupeptin (Sigma, #108976). Lysates were denatured for 5 min at 95°C in Laemmli buffer (1:1), loaded on 4–15% Tris HCl precast protein gels (BioRad, #345-0028), and resolved at 100 V for 2.5 hr. Proteins were transferred onto nitrocellulose (10 V overnight at 4°C; Amersham, #10600007). Membranes were blocked with 2.5% bovine serum albumin (Fisher, #1900-0016) in Tris-buffered saline containing 0.1% Tween-20 for 1 hr then incubated with primary antibody at 1:1000 overnight at 4 °C. Primary antibodies utilized were: phospho-AMPK (Thr172) (1:1000, Cell Signaling,#2535), total-AMPK (1:1000, Cell Signaling, #2532), phospho-mTOR (Ser 2448) (Cell Signaling, #5536), β-Actin (8H10D10) (1:1000, Cell Signaling, #3700).

Cortical neuron electrophysiology and live cell imaging
Mouse cortical neurons were prepared from E15 fetuses as described (9). Axion multielectrode plates (Axion Biosystems, # M768-tMEA-48B) were coated for 4 hr with 0.1% sterile polyethylenimine (Sigma Aldrich, #408727), washed 4 times with sterile water, and air dried overnight. Wells were incubated with 20 μg/mL laminin (Sigma Aldrich L2020) for 45 min at 37°C. Neurons were plated at 10^5 cells/well in 50 μL plate media (Dulbecco's modified Eagle medium (ThermoFisher, #11965092) with 10% FBS and 10% F12 supplement (Invitrogen 31765-035)). After 2 hr at 37°C, volume was brought to 500 μL/well with Neurobasal media (Invitrogen 21103-049) containing 2% B27 supplement (Invitrogen, #17504-044), 1% penicillin-streptomycin, and 0.5 mM Glutamax (ThermoFisher, #35050-061) supplemented with BDNF (10 ng/mL) (PeproTech, #450-02) and IGF-1 (10 ng/mL) (BD Biosciences, #354037). Half-media changes were performed every other day. Cortical neurons co-incubated with $CD8^+$ T cells were treated with 100 ng/ml IFNγ (R&D, #485-MI-100) for 24 hr prior to the addition of lymphocytes (10). $CD8^+$ OTI T cells were added at an E:T ratio of 2:1. For live cell imaging, neurons were plated on poly-ornithine coated (0.5 mg/mL; >4 hours at 37°C) microisolation chambers or 96 well glass bottom plates and transduced with AAV1.Syn.OVA-eGFP or AAV1.Syn.eGFP. Imaging was performed on an IncuCyte SX5 Liv-Cell Analysis System utilizing the built-in analysis platform to quantify EGFP+ area over time (normalized to baseline and reference controls). Imaging was initiated at 10-12 days in vitro. For electrophysiology, Axion multielectrode plates (Axion Biosystems, # M768-tMEA-48B) were coated for 4 hr with 0.1% sterile polyethylenimine (Sigma Aldrich, #408727), washed 4 times with sterile water, and air dried overnight. Wells were incubated with 20 μg/mL laminin (Sigma Aldrich L2020) for 45 min at 37°C. Realtime electrophysiological data were obtained starting on day 14-17 in vitro using the Axion Maestro platform. Spontaneous extracellular potentials were recorded for 10 minutes and wells with ≤ 2 active electrodes were excluded from the assay design. Active wells were divided into tiers according to overall spike number and then proportionately assigned to experimental conditions. Spontaneous firing was recorded for three 10-minute epochs at 37°C/5%CO2. Extracellular field potential recordings were collected every 10 minutes with a 10-minute rest between recordings over 18 hours. Filtered extracellular field potentials (Butterworth high pass at 200 Hz; lowpass at 3000 Hz) with unipolar peaks that exceeded 6 standard deviations (STD) above baseline as assessed by adaptive thresholding for each individual electrode were defined as spikes. Bursts were defined as ≥ 5 spikes on a single electrode with a maximum inter-spike interval of 100 msec and network bursts were defined as ≥ 50 spikes detected on ≥ 35% of electrodes/well with a maximum inter-spike interval of 100 msec.

scRNAseq Analysis
Compressed data from Gene Expression Omnibus series GSE138266 (11) were downloaded and unpacked to yield subject specific matrix, barcode, and gene files which were used to create Seurat objects in R (12, 13). The 6 MS patient-derived CSF scRNAseq samples and the 6 control CSF scRNAseq samples were merged into separate Seurat objects and normalized. After joining the layers in each object, variable features were identified, and a list of anchor genes was generated and used for integrating the two datasets. Data were

scaled, principal component analysis was performed, and dimension reduction was accomplished using uniform manifold approximation and projection. The shared nearest-neighbor graph was constructed with default Seurat parameters and used to determine clusters with the Louvain algorithm. Cell types in the resulting 16 clusters were further defined using ScType (14). Two $CD8^+$ clusters were identified and combined for subsequent analysis. Differentially expressed genes within the $CD8^+$ cluster between MS and controls were identified using the Wilcoxon rank sum test via the Presto fast implementation. The average log_2 fold change between MS and control and the Bonferroni-corrected P-value for the comparison were calculated and filtered on P<0.05. Selected target genes were queried and violin plots were generated.

Methods References

1. Clarkson BD, Johnson RK, Bingel C, Lothaller C, and Howe CL. Preservation of antigen-specific responses in cryopreserved CD4(+) and CD8(+) T cells expanded with IL-2 and IL-7. *J Transl Autoimmun.* 2022;5:100173.
2. Clarkson BD, Grund EM, Standiford MM, Mirchia K, Westphal MS, Muschler LS, et al. CD8+ T cells recognizing a neuron-restricted antigen injure axons in a model of multiple sclerosis. *J Clin Invest.* 2023;133(21).
3. Howe CL, LaFrance-Corey RG, Overlee BL, Johnson RK, Clarkson BDS, and Goddery EN. Inflammatory monocytes and microglia play independent roles in inflammatory ictogenesis. *J Neuroinflammation.* 2022;19(1):22.
4. Zhu A, Romero R, and Petty HR. An enzymatic colorimetric assay for glucose-6-phosphate. *Anal Biochem.* 2011;419(2):266-70.
5. Zerez CR, Lee SJ, and Tanaka KR. Spectrophotometric determination of oxidized and reduced pyridine nucleotides in erythrocytes using a single extraction procedure. *Anal Biochem.* 1987;164(2):367-73.
6. Lee HJ, Kremer DM, Sajjakulnukit P, Zhang L, and Lyssiotis CA. A large-scale analysis of targeted metabolomics data from heterogeneous biological samples provides insights into metabolite dynamics. *Metabolomics.* 2019;15(7):103.
7. Gonsalves WI, Ramakrishnan V, Hitosugi T, Ghosh T, Jevremovic D, Dutta T, et al. Glutamine-derived 2-hydroxyglutarate is associated with disease progression in plasma cell malignancies. *JCI Insight.* 2018;3(1).
8. Jennings ME, 2nd, and Matthews DE. Determination of complex isotopomer patterns in isotopically labeled compounds by mass spectrometry. *Anal Chem.* 2005;77(19):6435-44.
9. Clarkson BDS, Grund E, David K, Johnson RK, and Howe CL. ISGylation is induced in neurons by demyelination driving ISG15-dependent microglial activation. *J Neuroinflammation.* 2022;19(1):258.
10. Clarkson BDS, Patel MS, LaFrance-Corey RG, and Howe CL. Retrograde interferon-gamma signaling induces major histocompatibility class I expression in human-induced pluripotent stem cell-derived neurons. *Ann Clin Transl Neurol.* 2018;5(2):172-85.
11. Schafflick D, Xu CA, Hartlehnert M, Cole M, Schulte-Mecklenbeck A, Lautwein T, et al. Integrated single cell analysis of blood and cerebrospinal fluid leukocytes in multiple sclerosis. *Nat Commun.* 2020;11(1):247.
12. Hao Y, Hao S, Andersen-Nissen E, Mauck WM, 3rd, Zheng S, Butler A, et al. Integrated analysis of multimodal single-cell data. *Cell.* 2021;184(13):3573-87 e29.
13. Butler A, Hoffman P, Smibert P, Papalexi E, and Satija R. Integrating single-cell transcriptomic data across different conditions, technologies, and species. *Nat Biotechnol.* 2018;36(5):411-20.
14. Ianevski A, Giri AK, and Aittokallio T. Fully-automated and ultra-fast cell-type identification using specific marker combinations from single-cell transcriptomic data. *Nat Commun.* 2022;13(1):1246.

SUPPLEMENTAL FIGURES

Supplemental Figure 1.

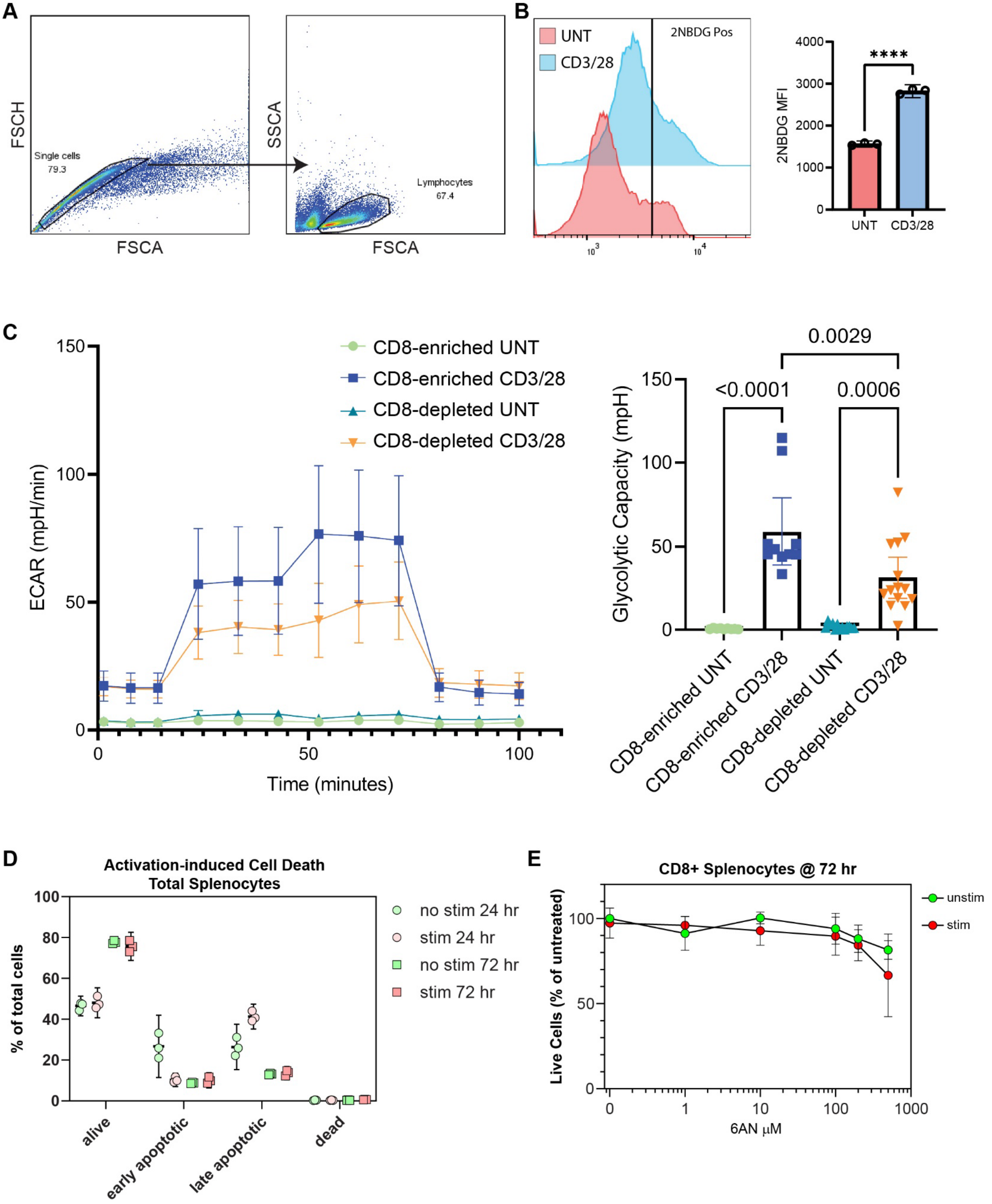

Supplemental Figure 1

(**A**) Gating strategy used to identify single cells and lymphocytes based on scatter profile. Gates were used for down-stream flow cytometry analyses. (**B**) Representative histograms showing that CD3/28 stimulation drove increased uptake of the fluorescent glucose analog 2-NBDG in splenocytes at 24 hr. The effect was significant by Mann Whitney test (n=3 per condition); **** $P<0.0001$. Unt = unstimulated; CD3/28 = stimulated with plate-bound CD3/CD28 antibodies for 24 hr. (**C**) Extracellular acidification rate (ECAR) is greater in CD8-enriched compared to CD8-depleted splenocytes. (left) ECAR measured using the Seahorse XF analyzer in magnetically-enriched or depleted splenocytes under non-activated (UNT) or CD3/28-activated (CD3/28) conditions at t = 24hrs; (right) quantification of glycolytic capacity, n=11-13/condition. (**D**) Mouse splenocytes were stimulated by plate-bound CD3/CD28 antibody or left unstimulated for 24 or 72 hours and cell death was assessed by flow cytometry. As expected, apoptotic cells were increased at 24 hr in the stimulated condition (early apoptotic: $P<0.0001$ by Tukey's pairwise comparison vs 24 hr unstimulated; late apoptotic: $P<0.0001$ by Tukey's pairwise comparison vs 24 hr unstimulated; alive or dead, not significant), consistent with activation-induced cell death. By 72 hr, there was no difference in any of the groups (alive, early apoptotic, late apoptotic, dead) between stimulated and unstimulated conditions ($F(9,32)=0.0001620$, $P>0.9999$ by two-way ANOVA for treatment effect; alive, unstim vs stim: $P=0.7225$; early apoptotic, unstim vs stim: $P=0.9049$; late apoptotic, unstim vs stim: $P=0.9939$; dead, unstim vs stim: $P=0.9989$; Tukey's multiple comparison). (**E**) 6AN was not toxic to CD8+ splenocytes through 100 μM (unstim, untreated vs 100 μM: $P=0.3155$; stim, untreated vs 100 μM: $P=0.2876$; Tukey's multiple comparison). Stimulation did not confer a significant survival effect for any dose of 6AN, relative to unstimulated ($F(1,24)=2.423$, $P=0.1327$ by two-way ANOVA).

Supplemental Figure 2

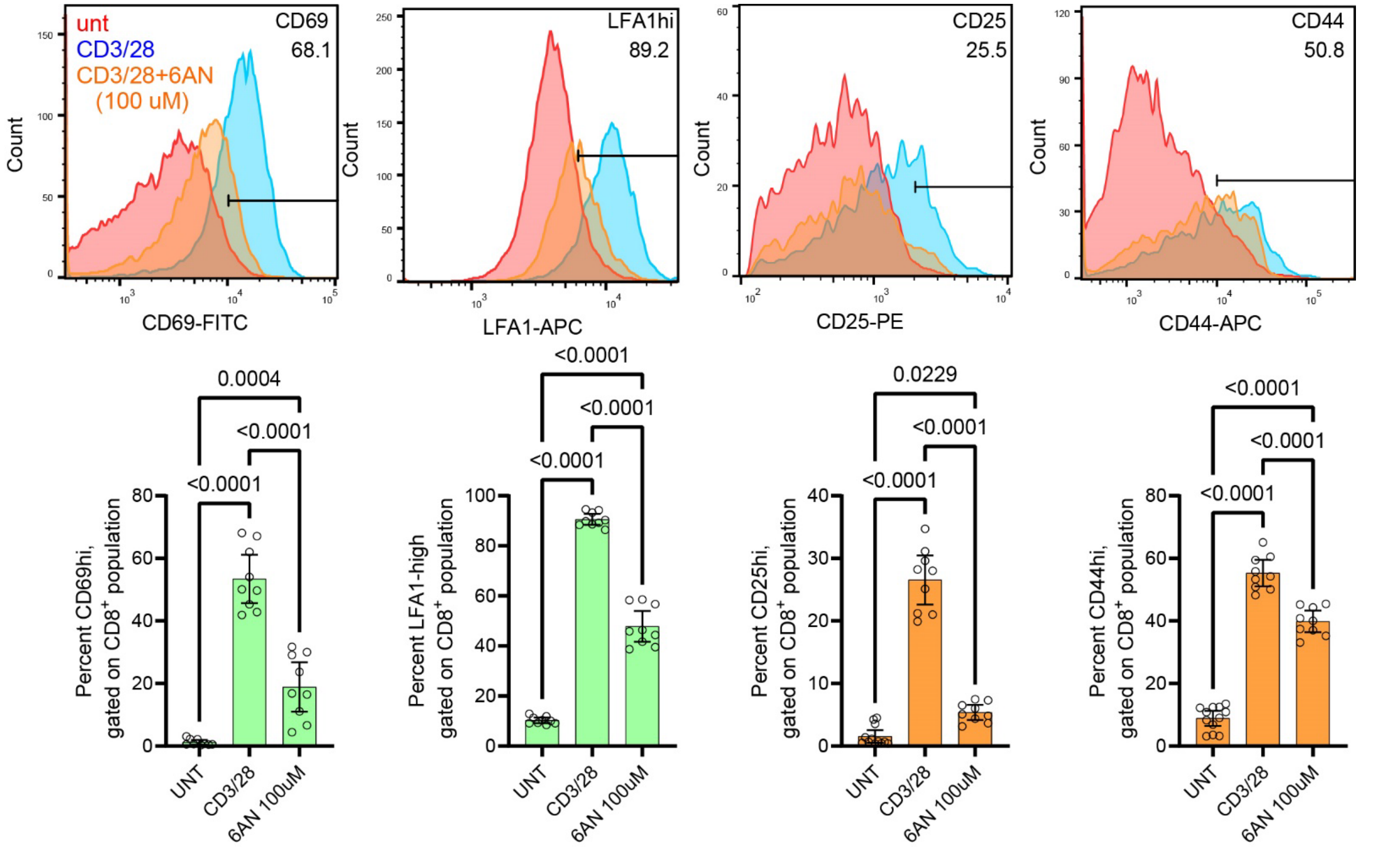

(Top) Representative histograms show gating strategy for surface activation markers including CD69, LFA-1, CD25, and CD44 on CD8$^+$ splenocytes 24 hr after treatment as indicated (unt = unstimulated; CD3/28 = anti-CD3/28 stimulated plus vehicle; CD3/28+6AN = anti-CD3/28 stimulated plus 6AN (100 μM). Percentages shown in the upper right quadrant represent CD3/28-stimulated plus vehicle condition. (Bottom) Quantitation of CD69, LFA-1, CD25, and CD44 expression on CD8$^+$ splenocytes 24 hr after treatment pooled from 3 separate experiments (N=3/condition; n=3/condition/experiment). Error bars are 95%CI; multiple comparison corrected P values are shown (one-way ANOVA with Tukey multiple comparison correction).

Supplemental Figure 3

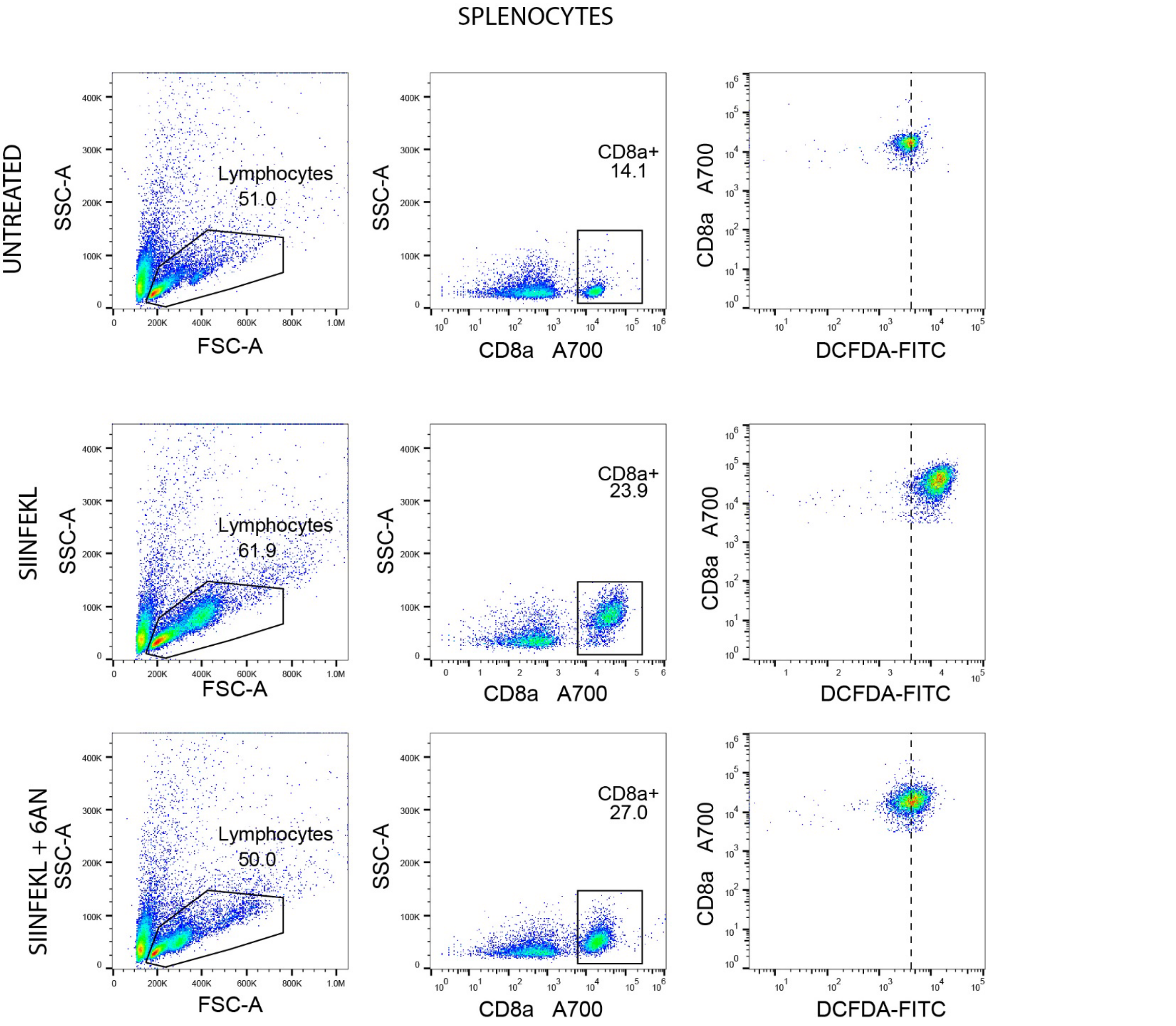

Gating strategy for measuring ROS production in OTI CD8$^+$ splenocytes 18 hours after stimulation with SIINFEKL in the absence or presence of 6AN (100 μM). Lymphocytes were gated on scatter profile and singlet discrimination. CD8$^+$ T cells were gated and intracellular ROS levels were quantified by measuring levels (MFI) of the redox-sensitive dye 2′,7′–dichlorofluorescein diacetate (DCFDA).

Supplemental Figure 4

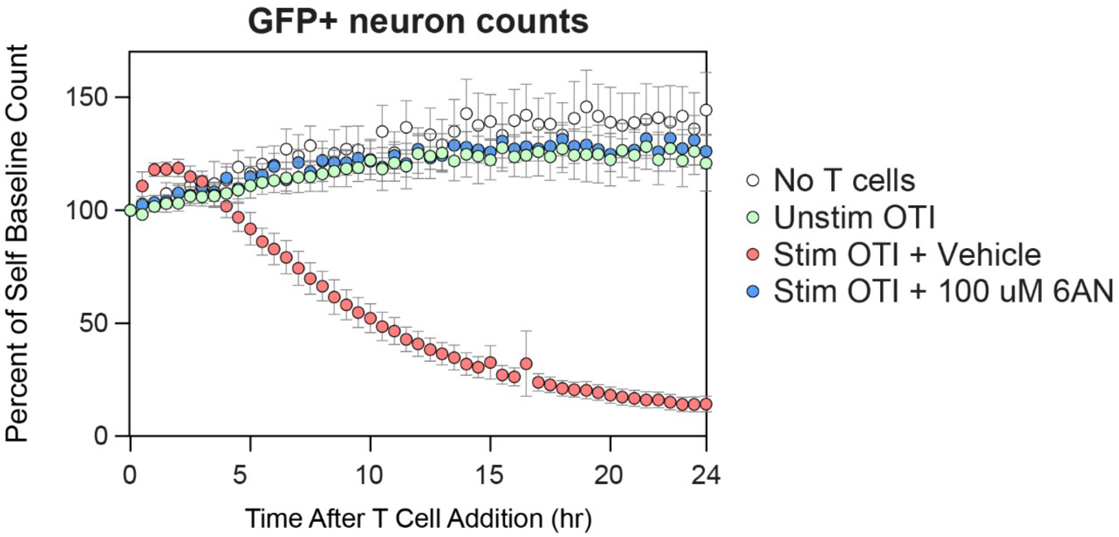

Live cell analysis of GFP+ neuron number calculated every 30 minutes over the first 24 hr after addition of unstimulated OTI T cells (n=15), CD3/28-stimulated OTI T cells pre-treated with vehicle (n=16), CD3/28-stimulated OTI T cells pre-treated with 6AN (100 μM) (n=15), or no T cells (n=13). Cell numbers were derived from the counting module on the Incucyte instrument. The number of GFP+ cells was significantly reduced in cultures co-incubated with stimulated OTI T cells pretreated with vehicle compared to cultures co-incubated with OTI T cells pretreated with 6AN by 5 hrs ($F(4,70)=102.2$, $P<0.0001$ for treatment effect by two-way ANOVA).

Supplemental Figure 5

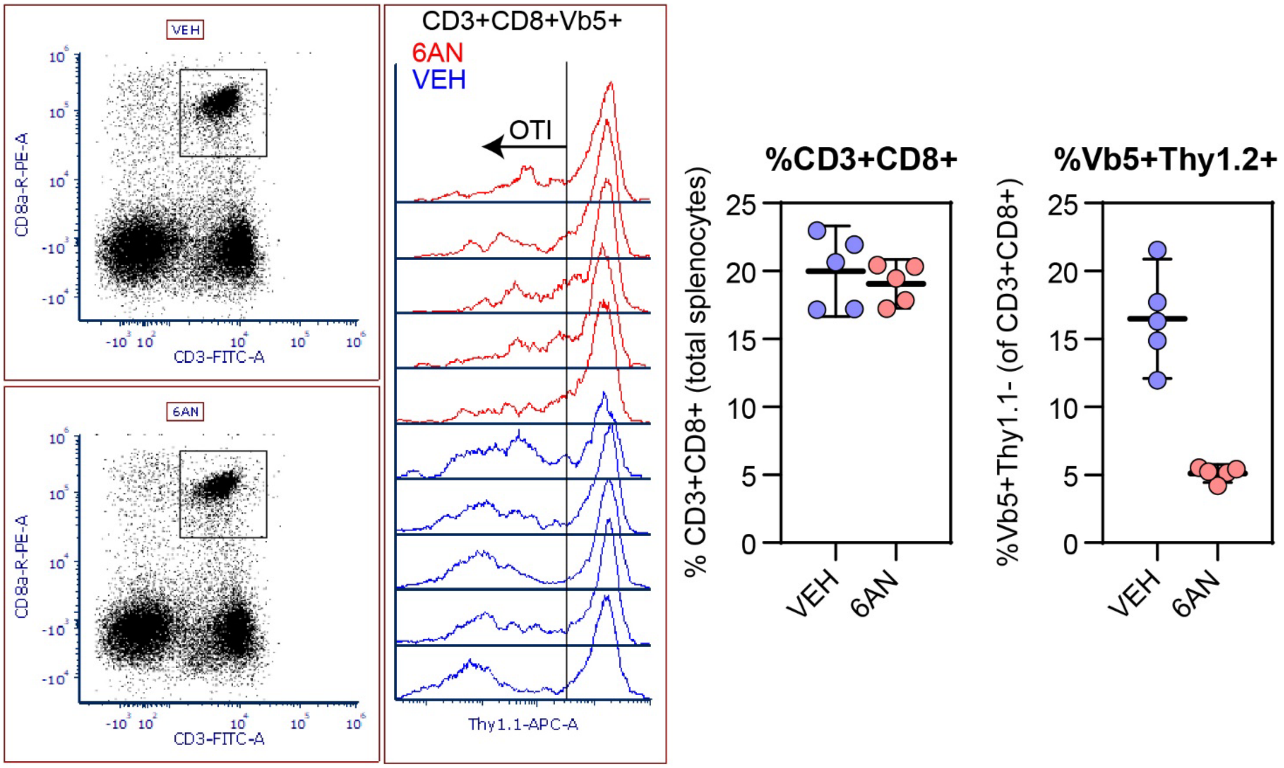

Analysis of Thy1.2^{+} (adoptively transferred) cells in the spleen of Thy1.1^{+} recipients at 8 days post-transfer. CD3^{+}CD8^{+} cells were gated in splenocytes from recipients receiving OTI splenocytes pretreated with vehicle (VEH) or pretreated with 6AN (100 μM) and then analyzed for level of Thy1.1 expression. While the percent of CD3^{+}CD8^{+} cells in the spleen did not differ between conditions (P=0.6905 by Mann Whitney), the percentage of these cells that were Vβ5.1/5.2^{+} and Thy1.1^{-} was reduced from about 15% in mice receiving OTI splenocytes pretreated with vehicle to about 5% in mice receiving OTI splenocytes pretreated with 6AN (P=0.0079 by Mann Whitney).

Supplemental Figure 6

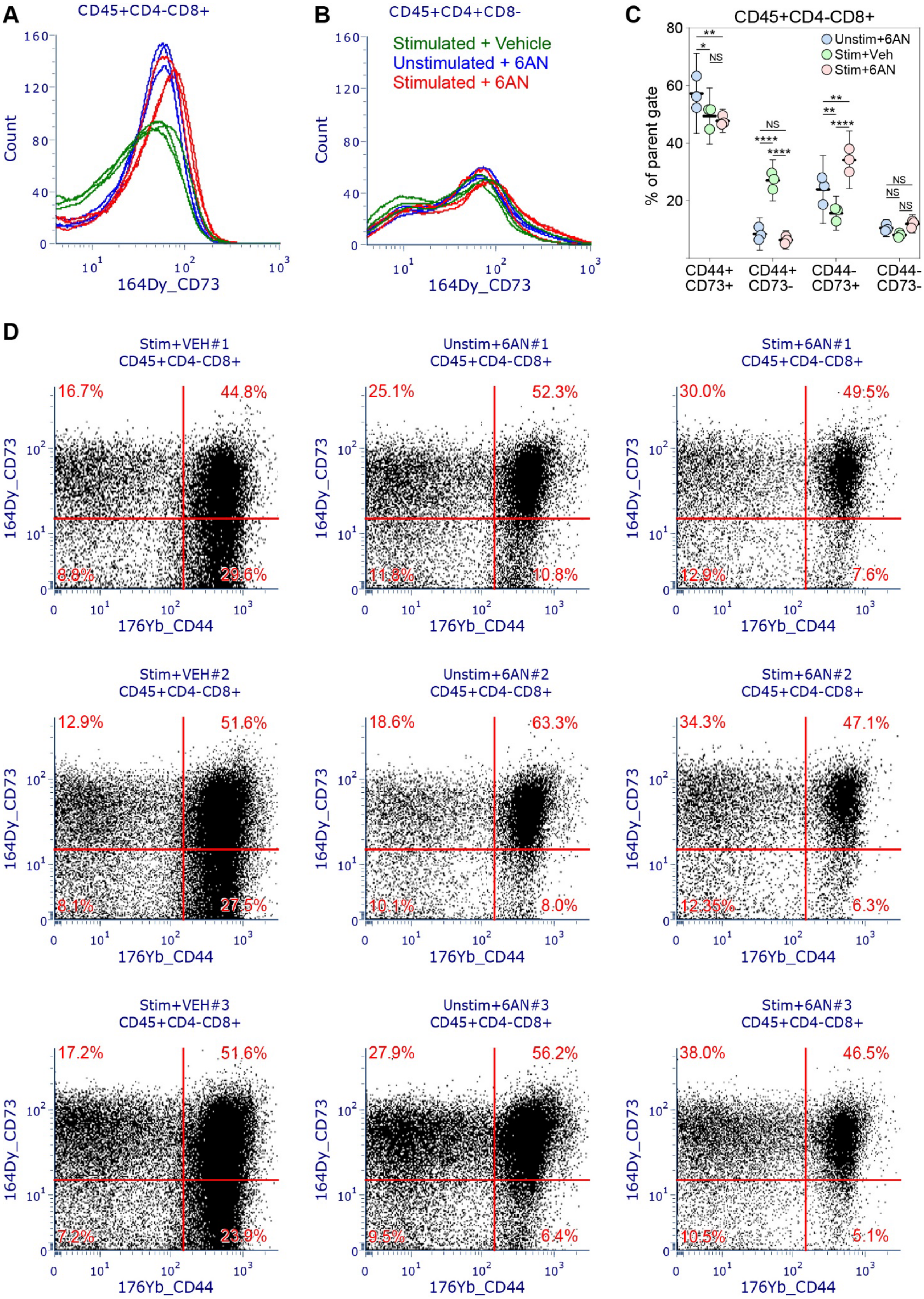

Supplemental Figure 6

(**A**) CD73 surface expression was reduced on cervical lymph node $CD45^{+}CD4^{-}CD8^{+}$ cells at 8 days post-transfer in mice receiving adoptive transfer of CD3/28-stimulated OTI splenocytes pre-treated with vehicle, relative to mice receiving adoptive transfer of CD3/28-stimulated OTI splenocytes pre-treated with 6AN or unstimulated OTI splenocytes pre-treated with 6AN (N=3 per condition). (**B**) This effect was not observed in $CD45^{+}CD4^{+}CD8^{+}$ cells in the cervical lymph nodes. (**C**) The effect of 6AN on CD73 downmodulation on $CD45^{+}CD4^{-}CD8^{+}$ cells was significant within the CD44- population, with compensatory changes in the percentage of cells in the $CD44^{+}CD73^{-}$ population. Error bars are 95%CI; * P<0.05, ** P<0.01, *** P<0.001, **** P<0.0001; NS = not significant by Tukey's pairwise analysis on two-way ANOVA. (**D**) Representative flow plots for the expression of CD73 and CD44 on $CD45^{+}CD4^{-}CD8^{+}$ cervical lymph node cells. Mice receiving stimulated OTI T cells pretreated with vehicle showed a large increase in a population of $CD73^{-}CD44^{+}$ cervical lymph node cells; this effect was blocked by treatment of the OTI cells with 6AN prior to adoptive transfer.

Supplemental Figure 7

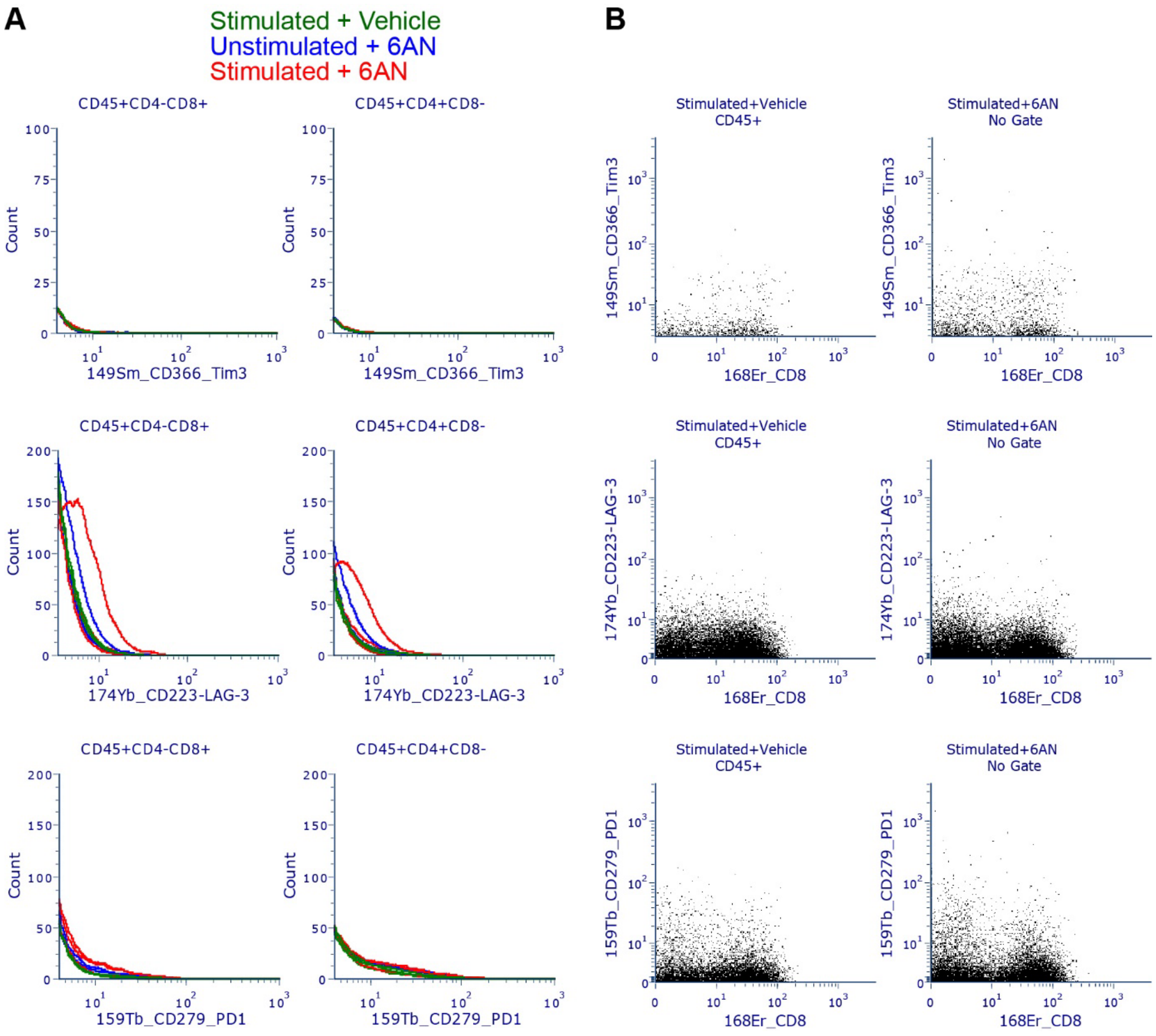

(**A**) Surface expression of TIM3, LAG3, and PD1 was not different on cervical lymph node $CD45^{+}CD4^{-}CD8^{+}$ cells at 8 days post-transfer in mice receiving adoptive transfer of CD3/28-stimulated OTI splenocytes pre-treated with vehicle, relative to mice receiving adoptive transfer of CD3/28-stimulated OTI splenocytes pre-treated with 6AN or unstimulated OTI splenocytes pre-treated with 6AN (N=3 per condition). (**B**) Representative flow plots for the expression of TIM3, LAG3, and PD1 on $CD45^{+}CD4^{-}CD8^{+}$ cervical lymph node cells.

Supplemental Figure 8

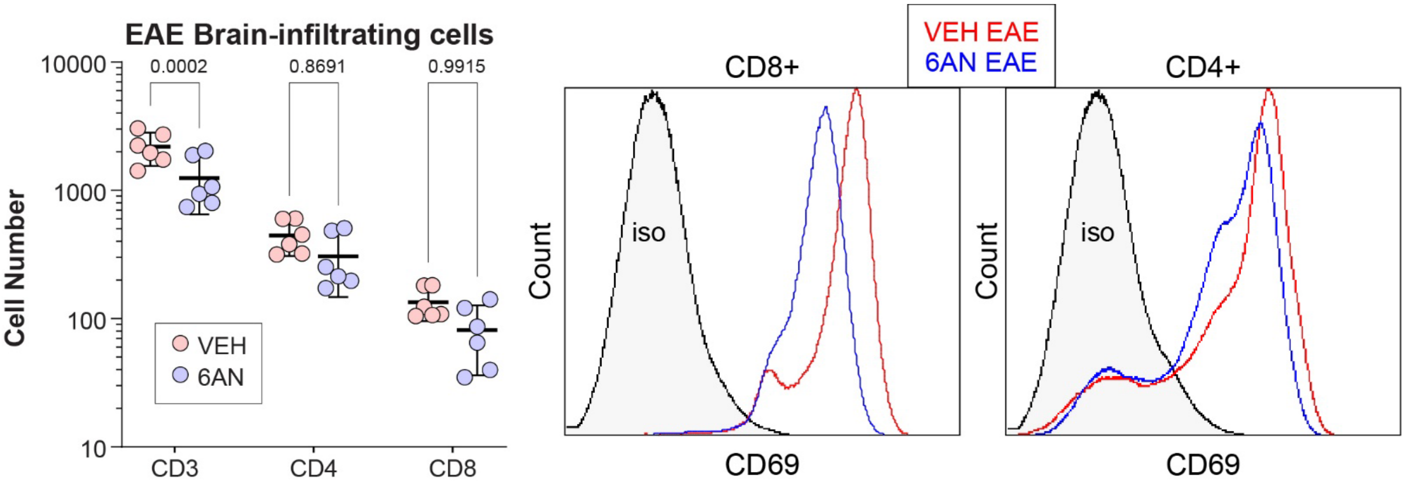

(Left) Brain-infiltrating cells were isolated 36 days after EAE induction from mice treated at 11, 13, 15, 17, 19 dpi with 6AN (5 mg/kg) (N=6) or with vehicle (N=6). Two-way ANOVA revealed a significant difference between treatment groups ($F(1,30)=10.48$, $P=0.0029$), with a significant difference in total $CD3^+$ cell numbers ($P=0.0002$ by Sidak's multiple comparison test) but no difference in the number of $CD4^+$ ($P=0.8691$) or $CD8^+$ subsets ($P=0.9915$). (Right) Representative histogram of CD69 expression level on $CD8^+$ or $CD4^+$ cells in the brain infiltrate at 28 dpi in mice treated with 6AN (blue) or vehicle (red).

SUPPLEMENTAL TABLE 1

2way ANOVA of G6P:ANOVA results

	Data Set-A	Data Set-B	Data Set-C	Data Set-D	Data Set-E
Table Analyzed	G6P				
Two-way ANOVA	Ordinary				
Alpha	0.05				
Source of Variation	% of total variation	P value	P value summary	Significant?	
Interaction	13.54	0.0042	**	Yes	
met_species	75.54	<0.0001	****	Yes	
Tx	2.975e-005	>0.9999	ns	No	
ANOVA table	SS	DF	MS	F (DFn, DFd)	P value
Interaction	2359	4	589.8	F (4, 18) = 5.575	P=0.0042
met_species	13167	2	6583	F (2, 18) = 62.23	P<0.0001
Tx	0.005185	2	0.002593	F (2, 18) = 2.451e-005	P>0.9999
Residual	1904	18	105.8		
Data summary					
Number of columns (Tx)	3				
Number of rows (met_species)	3				
Number of values	27				

2way ANOVA of G6P:Multiple comparisons

	Data Set-A	Data Set-B	Data Set-C	Data Set-D	Data Set-E	Data Set-F	Data Set-G	Data Set-H
Within each row, compare columns (simple effects within rows)								
	3							
Number of families	3							
Number of comparisons per family	0.05							

Alpha<br><br>Tukey's multiple comparisons test	Mean Diff.	95.00% CI of diff.	Below threshold?	Summary	Adjusted P Value			
m0								
UNT vs. ACT	12.80	-8.633 to 34.23	No	ns	0.3035			
UNT vs. ACT+6AN	-14.60	-36.03 to 6.833	No	ns	0.2185			
ACT vs. ACT+6AN	-27.40	-48.83 to -5.967	Yes	*	0.0114			
m1								
UNT vs. ACT	2.100	-19.33 to 23.53	No	ns	0.9662			
UNT vs. ACT+6AN	0.8333	-20.60 to 22.27	No	ns	0.9946			
ACT vs. ACT+6AN	-1.267	-22.70 to 20.17	No	ns	0.9875			
m2								
UNT vs. ACT	-14.87	-36.30 to 6.566	No	ns	0.2076			
UNT vs. ACT+6AN	13.70	-7.733 to 35.13	No	ns	0.2585			
ACT vs. ACT+6AN	28.57	7.134 to 50.00	Yes	**	0.0085			
Test details	Mean 1	Mean 2	Mean Diff.	SE of diff.	N1	N2	q	DF
m0						3	2.156	18.0
UNT vs. ACT	33.70	20.90	12.80		3	3	2.459	0
UNT vs. ACT+6AN	33.70	48.30	-14.60	8.398	3	3	4.614	18.0
ACT vs. ACT+6AN	20.90	48.30	-27.40	8.398	3			0
				8.398				18.0
m1						3	0.353	0
UNT vs. ACT	6.800	4.700	2.100		3	3	6	
UNT vs. ACT+6AN	6.800	5.967	0.8333	8.398	3	3	0.140	
ACT vs. ACT+6AN	4.700	5.967	-1.267	8.398	3		3	18.0
				8.398			0.213	0
m2						3	3	18.0
UNT vs. ACT	59.50	74.37	-14.87		3	3		0
UNT vs. ACT+6AN	59.50	45.80	13.70	8.398	3	3		18.0
ACT vs. ACT+6AN	74.37	45.80	28.57	8.398	3		2.504	0
				8.398			2.307	
							4.811	

								18.00<br>18.00<br>18.00

2way ANOVA of R5P:ANOVA results

	Data Set-A	Data Set-B	Data Set-C	Data Set-D	Data Set-E
Table Analyzed	R5P				
Two-way ANOVA	Ordinary				
Alpha	0.05				
Source of Variation	% of total variation	P value	P value summary	Significant?	
Interaction	19.39	<0.0001	****	Yes	
met_species	77.88	<0.0001	****	Yes	
Tx	6.593e-013	>0.9999	ns	No	
ANOVA table	SS	DF	MS	F (DFn, DFd)	P value
Interaction	2832	4	708.1	F (4, 18) = 31.99	P<0.0001
met_species	11375	2	5688	F (2, 18) = 256.9	P<0.0001
Tx	9.630e-011	2	4.815e-011	F (2, 18) = 2.175e-012	P>0.9999
Residual	398.4	18	22.14		
Data summary					
Number of columns (Tx)	3				
Number of rows (met_species)	3				
Number of values	27				

2way ANOVA of R5P:Multiple comparisons

	Data Set-A	Data Set-B	Data Set-C	Data Set-D	Data Set-E	Data Set-F	Data Set-G	Data Set-H
Within each row, compare columns (simple effects within rows)								
	3							
Number of families	3							
Number of comparisons per family	0.05							
Alpha								
Tukey's multiple comparisons test	Mean Diff.	95.00% CI of diff.	Below threshold?	Summary	Adjusted P Value			
m0								
UNT vs. ACT	20.81	11.00 to 30.61	Yes	***	0.0001			
UNT vs. ACT+6AN	-11.96	-21.76 to -2.154	Yes	*	0.0158			
ACT vs. ACT+6AN	-32.76	-42.57 to -22.96	Yes	****	<0.0001			
m1								
UNT vs. ACT	-21.23	-31.04 to -11.43	Yes	****	<0.0001			
UNT vs. ACT+6AN	1.827	-7.977 to 11.63	No	ns	0.8836			
ACT vs. ACT+6AN	23.06	13.26 to 32.86	Yes	****	<0.0001			
m2								
UNT vs. ACT	0.4282	-9.376 to 10.23	No	ns	0.9932			
UNT vs. ACT+6AN	10.13	0.3270 to 19.94	Yes	*	0.0422			
ACT vs. ACT+6AN	9.703	-0.1012 to 19.51	No	ns	0.0527			
						N2	q	DF
Test details	Mean 1	Mean 2	Mean Diff.	SE of diff.	N1			
m0						3	7.660	18.0
UNT vs. ACT	65.16	44.35	20.81		3	3	4.402	0
UNT vs. ACT+6AN	65.16	77.12	-11.96	3.841	3	3	12.06	18.0
ACT vs. ACT+6AN	44.35	77.12	-32.76	3.841	3			0
				3.841				18.0
m1						3	7.817	0
UNT vs. ACT	9.865	31.10	-21.23		3	3	0.672	

UNT vs. ACT+6AN	9.865	8.038	1.827	3.841	3	3	5	
ACT vs. ACT+6AN	31.10	8.038	23.06	3.841	3		8.490	18.0
				3.841				0
m2						3		18.0
UNT vs. ACT	24.98	24.55	0.4282		3	3	0.157	0
UNT vs. ACT+6AN	24.98	14.85	10.13	3.841	3	3	7	18.0
ACT vs. ACT+6AN	24.55	14.85	9.703	3.841	3		3.730	0
				3.841			3.572	
								18.0
								0
								18.0
								0
								18.0
								0

2way ANOVA of S7P:ANOVA results

	Data Set-A	Data Set-B	Data Set-C	Data Set-D	Data Set-E
Table Analyzed	S7P				
Two-way ANOVA	Ordinary				
Alpha	0.05				
Source of Variation	% of total variation	P value	P value summary	Significant?	
Interaction	16.97	<0.0001	****	Yes	
met_species	82.55	<0.0001	****	Yes	
Tx	5.756e-012	>0.9999	ns	No	
ANOVA table	SS	DF	MS	F (DFn, DFd)	P value
Interaction	2140	4	535.0	F (4, 18) = 159.0	P<0.0001
met_species	10412	2	5206	F (2, 18) = 1547	P<0.0001
Tx	7.260e-010	2	3.630e-010	F (2, 18) = 1.079e-010	P>0.9999
Residual	60.58	18	3.365		
Data summary					

Number of columns (Tx)	3				
Number of rows (met_species)	3				
Number of values	27				

2way ANOVA of S7P:Multiple comparisons

	Data Set-A	Data Set-B	Data Set-C	Data Set-D	Data Set-E	Data Set-F	Data Set-G	Data Set-H
Within each row, compare columns (simple effects within rows)								
	3							
Number of families	3							
Number of comparisons per family	0.05							
Alpha								
Tukey's multiple comparisons test	Mean Diff.	95.00% CI of diff.	Below threshold?	Summary	Adjusted P Value			
m0								
UNT vs. ACT	23.86	20.04 to 27.68	Yes	****	<0.0001			
UNT vs. ACT+6AN	-4.099	-7.922 to -	Yes	*	0.0345			
ACT vs. ACT+6AN	-27.96	0.2761	Yes	****	<0.0001			
		-31.78 to -24.13						
m1								
UNT vs. ACT	-15.75		Yes	****	<0.0001			
UNT vs. ACT+6AN	3.486	-19.57 to -11.93	No	ns	0.0774			
ACT vs. ACT+6AN	19.24	-0.3363 to 7.309	Yes	****	<0.0001			
		15.41 to 23.06						
m2								
UNT vs. ACT	-8.108		Yes	***	0.0001			
UNT vs. ACT+6AN	0.6125	-11.93 to -4.286	No	ns	0.9124			
ACT vs. ACT+6AN	8.721	-3.210 to 4.435	Yes	****	<0.0001			
		4.898 to 12.54						
Test details	Mean 1		Mean Diff.	SE of	N1	N2	q	DF

		Mean 2		diff.				
m0						3	22.53	18.0
UNT vs. ACT	67.56		23.86		3	3	3.870	0
UNT vs. ACT+6AN	67.56	43.70	-4.099	1.498	3	3	26.40	18.0
ACT vs. ACT+6AN	43.70	71.65	-27.96	1.498	3			0
		71.65		1.498				18.0
m1						3	14.87	0
UNT vs. ACT	13.05		-15.75		3	3	3.292	
UNT vs. ACT+6AN	13.05	28.80	3.486	1.498	3	3	18.16	
ACT vs. ACT+6AN	28.80	9.566	19.24	1.498	3			18.0
		9.566		1.498				0
m2						3	7.656	18.0
UNT vs. ACT	19.39		-8.108		3	3	0.578	0
UNT vs. ACT+6AN	19.39	27.50	0.6125	1.498	3	3	3	18.0
ACT vs. ACT+6AN	27.50	18.78	8.721	1.498	3		8.234	0
		18.78		1.498				
								18.0
								0
								18.0
								0
								18.0
								0

2way ANOVA of F6P:ANOVA results

	Data Set-A	Data Set-B	Data Set-C	Data Set-D	Data Set-E
Table Analyzed	F6P				
Two-way ANOVA	Ordinary				
Alpha	0.05				
Source of Variation	% of total variation	P value	P value summary	Significant?	
Interaction	23.80	<0.0001	****	Yes	
met_species	70.59	<0.0001	****	Yes	
Tx	3.719e-011	>0.9999	ns	No	

ANOVA table	SS	DF	MS	F (DFn, DFd)	P value
Interaction	1495	4	373.6	F (4, 18) = 19.10	P<0.0001
met_species	4433	2	2216	F (2, 18) = 113.3	P<0.0001
Tx	2.336e-009	2	1.168e-009	F (2, 18) = 5.969e-011	P>0.9999
Residual	352.2	18	19.57		
Data summary					
Number of columns (Tx)	3				
Number of rows (met_species)	3				
Number of values	27				

2way ANOVA of F6P:Multiple comparisons

	Data Set-A	Data Set-B	Data Set-C	Data Set-D	Data Set-E	Data Set-F	Data Set-G	Data Set-H
Within each row, compare columns (simple effects within rows)								
	3							
Number of families	3							
Number of comparisons per family	0.05							
Alpha								
Tukey's multiple comparisons test	Mean Diff.	95.00% CI of diff.	Below threshold?	Summary	Adjusted P Value			
m0								
UNT vs. ACT	15.85	6.636 to 25.07	Yes	***	0.0010			
UNT vs. ACT+6AN	-8.581	-17.80 to 0.6360	No	ns	0.0705			
ACT vs. ACT+6AN	-24.43	-33.65 to -15.22	Yes	****	<0.0001			
m1								
UNT vs. ACT	-13.24	-22.46 to -4.024	Yes	**	0.0048			
UNT vs. ACT+6AN	4.220	-4.997 to 13.44	No	ns	0.4863			
ACT vs. ACT+6AN	17.46	8.244 to 26.68	Yes	***	0.0004			

m2								
UNT vs. ACT	-2.612	-11.83 to 6.606	No	ns	0.7532			
UNT vs. ACT+6AN	4.361	-4.856 to 13.58	No	ns	0.4640			
ACT vs. ACT+6AN	6.973	-2.244 to 16.19	No	ns	0.1589			
						N2	q	DF
Test details	Mean 1	Mean 2	Mean Diff.	SE of diff.	N1			
m0						3	6.20	18.0
UNT vs. ACT	42.01	26.16	15.85		3	3	8	0
UNT vs. ACT+6AN	42.01	50.59	-8.581	3.612	3	3	3.36	18.0
ACT vs. ACT+6AN	26.16	50.59	-24.43	3.612	3		0	0
				3.612			9.56	18.0
m1						3	8	0
UNT vs. ACT	12.47	25.71	-13.24		3	3		
UNT vs. ACT+6AN	12.47	8.250	4.220	3.612	3	3		
ACT vs. ACT+6AN	25.71	8.250	17.46	3.612	3		5.18	18.0
				3.612			5	0
m2						3	1.65	18.0
UNT vs. ACT	45.52	48.13	-2.612		3	3	3	0
UNT vs. ACT+6AN	45.52	41.16	4.361	3.612	3	3	6.83	18.0
ACT vs. ACT+6AN	48.13	41.16	6.973	3.612	3		8	0
				3.612				
							1.02	18.0
							3	0
							1.70	18.0
							8	0
							2.73	18.0
							0	0

2way ANOVA of Ru5P:ANOVA results

	Data Set-A	Data Set-B	Data Set-C	Data Set-D	Data Set-E

Table Analyzed	Ru5P				
Two-way ANOVA	Ordinary				
Alpha	0.05				
Source of Variation	% of total variation	P value	P value summary	Significant?	
Interaction	6.705	<0.0001	****	Yes	
met_species	93.23	<0.0001	****	Yes	
Tx	2.767e-012	>0.9999	ns	No	
ANOVA table	SS	DF	MS	F (DFn, DFd)	P value
Interaction	2325	4	581.2	F (4, 18) = 433.0	P<0.0001
met_species	32325	2	16163	F (2, 18) = 12041	P<0.0001
Tx	9.593e-010	2	4.796e-010	F (2, 18) = 3.573e-010	P>0.9999
Residual	24.16	18	1.342		
Data summary					
Number of columns (Tx)	3				
Number of rows (met_species)	3				
Number of values	27				

2way ANOVA of Ru5P:Multiple comparisons

	Data Set-A	Data Set-B	Data Set-C	Data Set-D	Data Set-E	Data Set-F	Data Set-G	Data Set-H
Within each row, compare columns (simple effects within rows)								
	3							
Number of families	3							
Number of comparisons per family	0.05							
Alpha								
	Mean Diff.	95.00% CI of diff.	Below threshold?	Summary	Adjusted P Value			
Tukey's multiple comparisons test								

m0								
UNT vs. ACT	26.06	23.65 to 28.48	Yes	****	<0.0001			
UNT vs. ACT+6AN	0.9401	-1.474 to 3.354	No	ns	0.5900			
ACT vs. ACT+6AN	-25.12	-27.54 to -22.71	Yes	****	<0.0001			
m1								
UNT vs. ACT	-21.27	-23.68 to -18.85	Yes	****	<0.0001			
UNT vs. ACT+6AN	1.621	-0.7931 to 4.035	No	ns	0.2274			
ACT vs. ACT+6AN	22.89	20.48 to 25.30	Yes	****	<0.0001			
m2								
UNT vs. ACT	-4.794	-7.208 to -2.379	Yes	***	0.0002			
UNT vs. ACT+6AN	-2.561	-4.976 to -	Yes	*	0.0366			
ACT vs. ACT+6AN	2.232	0.1470	No	ns	0.0728			
		-0.1820 to 4.647						
						N2	q	DF
Test details	Mean 1		Mean Diff.	SE of	N1			
		Mean 2		diff.				
m0						3	38.9	18.0
UNT vs. ACT	90.94		26.06		3	3	6	0
UNT vs. ACT+6AN	90.94	64.88	0.9401	0.9460	3	3	1.40	18.0
ACT vs. ACT+6AN	64.88	90.00	-25.12	0.9460	3		5	0
		90.00		0.9460			37.5	18.0
m1						3	6	0
UNT vs. ACT	7.392		-21.27		3	3		
UNT vs. ACT+6AN	7.392	28.66	1.621	0.9460	3	3		
ACT vs. ACT+6AN	28.66	5.771	22.89	0.9460	3		31.8	18.0
		5.771		0.9460			0	0
m2						3	2.42	18.0
UNT vs. ACT	1.670		-4.794		3	3	4	0
UNT vs. ACT+6AN	1.670	6.464	-2.561	0.9460	3	3	34.2	18.0
ACT vs. ACT+6AN	6.464	4.232	2.232	0.9460	3		2	0
		4.232		0.9460				
							7.16	18.0
							6	0
							3.82	18.0
							9	0

							3.337	18.00

2way ANOVA of G3P:ANOVA results

	Data Set-A	Data Set-B	Data Set-C	Data Set-D	Data Set-E
Table Analyzed	G3P				
Two-way ANOVA	Ordinary				
Alpha	0.05				
Source of Variation	% of total variation	P value	P value summary	Significant?	
Interaction	0.1443	<0.0001	****	Yes	
met_species	99.82	<0.0001	****	Yes	
Tx	9.908e-013	>0.9999	ns	No	
ANOVA table	SS	DF	MS	F (DFn, DFd)	P value
Interaction	76.96	4	19.24	F (4, 18) = 20.44	P<0.0001
met_species	53225	2	26612	F (2, 18) = 28273	P<0.0001
Tx	5.283e-010	2	2.641e-010	F (2, 18) = 2.806e-010	P>0.9999
Residual	16.94	18	0.9413		
Data summary					
Number of columns (Tx)	3				
Number of rows (met_species)	3				
Number of values	27				

2way ANOVA of G3P:Multiple comparisons

	Data Set-A	Data Set-B	Data Set-C	Data Set-D	Data Set-E	Data Set-F	Data Set-G	Data Set-H

Within each row, compare columns (simple effects within rows)								
	3							
Number of families	3							
Number of comparisons per family	0.05							
Alpha								
Tukey's multiple comparisons test	Mean Diff.	95.00% CI of diff.	Below threshold?	Summary	Adjusted P Value			
m0								
UNT vs. ACT	1.516	-0.5061 to 3.537	No	ns	0.1637			
UNT vs. ACT+6AN	-3.427	-5.449 to -1.406	Yes	**	0.0011			
ACT vs. ACT+6AN	-4.943	-6.965 to -2.921	Yes	****	<0.0001			
m1								
UNT vs. ACT	-1.516	-3.537 to 0.5061	No	ns	0.1637			
UNT vs. ACT+6AN	3.427	1.406 to 5.449	Yes	**	0.0011			
ACT vs. ACT+6AN	4.943	2.921 to 6.965	Yes	****	<0.0001			
m2								
UNT vs. ACT	0.000	-2.022 to 2.022	No	ns	>0.9999			
UNT vs. ACT+6AN	0.000	-2.022 to 2.022	No	ns	>0.9999			
ACT vs. ACT+6AN	0.000	-2.022 to 2.022	No	ns	>0.9999			
Test details	Mean 1	Mean 2	Mean Diff.	SE of diff.	N1	N2	q	DF
m0						3	2.70	18.0
UNT vs. ACT	95.45	93.93	1.516		3	3	6	0
UNT vs. ACT+6AN	95.45	98.87	-3.427	0.7922	3	3	6.11	18.0
ACT vs. ACT+6AN	93.93	98.87	-4.943	0.7922	3		9	0
				0.7922			8.82	18.0
m1						3	5	0
UNT vs. ACT	4.555	6.070	-1.516		3	3		
UNT vs. ACT+6AN	4.555	1.127	3.427	0.7922	3	3		
ACT vs. ACT+6AN	6.070	1.127	4.943	0.7922	3		2.70	18.0
				0.7922			6	0
m2						3	6.11	18.0

UNT vs. ACT<br>UNT vs. ACT+6AN<br>ACT vs. ACT+6AN	0.000<br>0.000<br>0.000	0.000<br>0.000<br>0.000	0.000<br>0.000<br>0.000	 0.7922<br>0.7922<br>0.7922	3<br>3<br>3	3<br>3	9<br>8.82<br>5<br><br><br>0.00<br>0<br>0.00<br>0<br>0.00<br>0	0<br>18.0<br>0<br><br><br>18.0<br>0<br>18.0<br>0<br>18.0<br>0

2way ANOVA of X5P:ANOVA results

	Data Set-A	Data Set-B	Data Set-C	Data Set-D	Data Set-E
Table Analyzed	X5P				
Two-way ANOVA	Ordinary				
Alpha	0.05				
Source of Variation	% of total variation	P value	P value summary	Significant?	
Interaction	9.438	<0.0001	****	Yes	
met_species	90.55	<0.0001	****	Yes	
Tx	4.669e-014	>0.9999	ns	No	
ANOVA table	SS	DF	MS	F (DFn, DFd)	P value
Interaction	3369	4	842.3	F (4, 18) = 4153	P<0.0001
met_species	32326	2	16163	F (2, 18) = 79695	P<0.0001
Tx	1.667e-011	2	8.333e-012	F (2, 18) = 4.109e-011	P>0.9999
Residual	3.651	18	0.2028		
Data summary					
Number of columns (Tx)	3				
Number of rows (met_species)	3				
Number of values	27				

2way ANOVA of X5P:Multiple comparisons

	Data Set-A	Data Set-B	Data Set-C	Data Set-D	Data Set-E	Data Set-F	Data Set-G	Data Set-H
Within each row, compare columns (simple effects within rows)								
	3							
Number of families	3							
Number of comparisons per family	0.05							
Alpha								
Tukey's multiple comparisons test	Mean Diff.	95.00% CI of diff.	Below threshold?	Summary	Adjusted P Value			
m0								
UNT vs. ACT	26.33	25.39 to 27.27	Yes	****	<0.0001			
UNT vs. ACT+6AN	-4.789	-5.727 to -3.850	Yes	****	<0.0001			
ACT vs. ACT+6AN	-31.12	-32.06 to -30.18	Yes	****	<0.0001			
m1								
UNT vs. ACT	-26.33	-27.27 to -25.39	Yes	****	<0.0001			
UNT vs. ACT+6AN	4.789	3.850 to 5.727	Yes	****	<0.0001			
ACT vs. ACT+6AN	31.12	30.18 to 32.06	Yes	****	<0.0001			
m2								
UNT vs. ACT	0.000	-0.9385 to	No	ns	>0.9999			
UNT vs. ACT+6AN	0.000	0.9385	No	ns	>0.9999			
ACT vs. ACT+6AN	0.000	-0.9385 to	No	ns	>0.9999			
		0.9385						
		-0.9385 to				N2	q	DF
Test details	Mean 1	0.9385	Mean Diff.	SE of diff.	N1			
m0						3	101.	18.0
UNT vs. ACT	88.21	Mean 2	26.33		3	3	3	0
UNT vs. ACT+6AN	88.21		-4.789	0.3677	3	3	18.4	18.0
ACT vs. ACT+6AN	61.88		-31.12	0.3677	3		2	0

		61.88		0.3677			119.	18.0
m1		92.99				3	7	0
UNT vs. ACT	11.79	92.99	-26.33		3	3		
UNT vs. ACT+6AN	11.79		4.789	0.3677	3	3		
ACT vs. ACT+6AN	38.12		31.12	0.3677	3		101.	18.0
		38.12		0.3677			3	0
m2		7.005				3	18.4	18.0
UNT vs. ACT	0.000	7.005	0.000		3	3	2	0
UNT vs. ACT+6AN	0.000		0.000	0.3677	3	3	119.	18.0
ACT vs. ACT+6AN	0.000		0.000	0.3677	3		7	0
		0.000		0.3677				
		0.000						
		0.000					0.00	18.0
							0	0
							0.00	18.0
							0	0
							0.00	18.0
							0	0

2way ANOVA of DHAP:ANOVA results

	Data Set-A	Data Set-B	Data Set-C	Data Set-D	Data Set-E
Table Analyzed	DHAP				
Two-way ANOVA	Ordinary				
Alpha	0.05				
Source of Variation	% of total variation	P value	P value summary	Significant?	
Interaction	0.06549	<0.0001	****	Yes	
met_species	99.93	<0.0001	****	Yes	
Tx	4.668e-013	>0.9999	ns	No	
ANOVA table	SS	DF	MS	F (DFn, DFd)	P value
Interaction	34.08	4	8.519	F (4, 18) = 85.39	P<0.0001
met_species	51999	2	25999	F (2, 18) = 260621	P<0.0001
Tx	2.429e-010			F (2, 18) = 1.217e-009	P>0.9999

Residual<br><br>Data summary<br>Number of columns (Tx)<br>Number of rows (met_species)<br>Number of values	1.796<br><br><br>3<br>3<br>27	2<br>18	1.214e-010<br>0.09976		

2way ANOVA of DHAP:Multiple comparisons

	Data Set-A	Data Set-B	Data Set-C	Data Set-D	Data Set-E	Data Set-F	Data Set-G	Data Set-H
Within each row, compare columns (simple effects within rows)<br><br>Number of families<br>Number of comparisons per family<br>Alpha	 <br>3<br>3<br>0.05							
Tukey's multiple comparisons test	Mean Diff.	95.00% CI of diff.	Below threshold?	Summary	Adjusted P Value			
m0<br>UNT vs. ACT<br>UNT vs. ACT+6AN<br>ACT vs. ACT+6AN	 2.356<br>-0.9090<br>-3.265	 1.698 to 3.014<br>-1.567 to -<br>0.2508<br>-3.923 to -2.607	 Yes<br>Yes<br>Yes	 ****<br>**<br>****	 <0.0001<br>0.0065<br><0.0001			
m1<br>UNT vs. ACT<br>UNT vs. ACT+6AN<br>ACT vs. ACT+6AN	 -2.356<br>0.9090<br>3.265	 <br>-3.014 to -1.698<br>0.2509 to 1.567<br>2.607 to 3.923	 Yes<br>Yes<br>Yes	 ****<br>**<br>****	 <0.0001<br>0.0065<br><0.0001			
m2<br>UNT vs. ACT<br>UNT vs. ACT+6AN<br>ACT vs. ACT+6AN	 0.000<br>0.000<br>0.000	 <br>-0.6582 to<br>0.6582	 No<br>No<br>No	 ns<br>ns<br>ns	 >0.9999<br>>0.9999<br>>0.9999			

		-0.6582 to						
		0.6582				N2	q	DF
Test details	Mean 1	-0.6582 to	Mean Diff.	SE of	N1			
		0.6582		diff.				
m0						3	12.9	18.0
UNT vs. ACT	95.82		2.356		3	3	2	0
UNT vs. ACT+6AN	95.82	Mean 2	-0.9090	0.2579	3	3	4.98	18.0
ACT vs. ACT+6AN	93.46		-3.265	0.2579	3		5	0
				0.2579			17.9	18.0
m1		93.46				3	0	0
UNT vs. ACT	4.180	96.73	-2.356		3	3		
UNT vs. ACT+6AN	4.180	96.73	0.9090	0.2579	3	3		
ACT vs. ACT+6AN	6.536		3.265	0.2579	3		12.9	18.0
				0.2579			2	0
m2		6.536				3	4.98	18.0
UNT vs. ACT	0.000	3.271	0.000		3	3	5	0
UNT vs. ACT+6AN	0.000	3.271	0.000	0.2579	3	3	17.9	18.0
ACT vs. ACT+6AN	0.000		0.000	0.2579	3		0	0
				0.2579				
		0.000						
		0.000					0.00	18.0
		0.000					0	0
							0.00	18.0
							0	0
							0.00	18.0
							0	0

2way ANOVA of 6PG:ANOVA results

	Data Set-A	Data Set-B	Data Set-C	Data Set-D	Data Set-E
Table Analyzed	6PG				
Two-way ANOVA	Ordinary				
Alpha	0.05				
Source of Variation	% of total variation	P value	P value summary	Significant?	

Interaction	0.1217	<0.0001	****	Yes	
met_species	99.85	<0.0001	****	Yes	
Tx	5.012e-014	>0.9999	ns	No	
ANOVA table	SS	DF	MS	F (DFn, DFd)	P value
Interaction	58.81	4	14.70	F (4, 18) = 20.60	P<0.0001
met_species	48262	2	24131	F (2, 18) = 33808	P<0.0001
Tx	2.423e-011	2	1.211e-011	F (2, 18) = 1.697e-011	P>0.9999
Residual	12.85	18	0.7138		
Data summary					
Number of columns (Tx)	3				
Number of rows (met_species)	3				
Number of values	27				

2way ANOVA of 6PG:Multiple comparisons

	Data Set-A	Data Set-B	Data Set-C	Data Set-D	Data Set-E	Data Set-F	Data Set-G	Data Set-H
Within each row, compare columns (simple effects within rows)								
	3							
Number of families	3							
Number of comparisons per family	0.05							
Alpha								
Tukey's multiple comparisons test	Mean Diff.	95.00% CI of diff.	Below threshold?	Summary	Adjusted P Value			
m0								
UNT vs. ACT	4.052	2.291 to 5.812	Yes	****	<0.0001			
UNT vs. ACT+6AN	3.572	1.811 to 5.332	Yes	***	0.0002			
ACT vs. ACT+6AN	-0.4801	-2.241 to 1.280	No	ns	0.7688			
m1								

UNT vs. ACT	-4.052	-5.812 to -2.291	Yes	****	<0.0001			
UNT vs. ACT+6AN	-3.572	-5.332 to -1.811	Yes	***	0.0002			
ACT vs. ACT+6AN	0.4801	-1.280 to 2.241	No	ns	0.7688			
m2								
UNT vs. ACT	0.000	-1.761 to 1.761	No	ns	>0.9999			
UNT vs. ACT+6AN	0.000	-1.761 to 1.761	No	ns	>0.9999			
ACT vs. ACT+6AN	0.000	-1.761 to 1.761	No	ns	>0.9999			
						N2	q	DF
Test details	Mean 1	Mean 2	Mean Diff.	SE of diff.	N1			
m0						3	8.307	18.0
UNT vs. ACT	95.53	91.48	4.052		3	3	7.322	0
UNT vs. ACT+6AN	95.53	91.96	3.572	0.6898	3	3	0.984	18.0
ACT vs. ACT+6AN	91.48	91.96	-0.4801	0.6898	3		3	0
				0.6898				18.0
m1						3		0
UNT vs. ACT	4.472	8.524	-4.052		3	3	8.307	
UNT vs. ACT+6AN	4.472	8.043	-3.572	0.6898	3	3	7.322	
ACT vs. ACT+6AN	8.524	8.043	0.4801	0.6898	3		0.984	18.0
				0.6898			3	0
m2						3		18.0
UNT vs. ACT	0.000	0.000	0.000		3	3		0
UNT vs. ACT+6AN	0.000	0.000	0.000	0.6898	3	3	0.000	18.0
ACT vs. ACT+6AN	0.000	0.000	0.000	0.6898	3		0.000	0
				0.6898			0.000	
								18.0
								0
								18.0
								0
								18.0
								0

2way ANOVA of 2PG:ANOVA results

	Data Set-A	Data Set-B	Data Set-C	Data Set-D	Data Set-E
Table Analyzed	2PG				
Two-way ANOVA	Ordinary				
Alpha	0.05				
Source of Variation	% of total variation	P value	P value summary	Significant?	
Interaction	0.04672	0.0002	***	Yes	
met_species	99.93	<0.0001	****	Yes	
Tx	6.059e-013	>0.9999	ns	No	
ANOVA table	SS	DF	MS	F (DFn, DFd)	P value
Interaction	23.00	4	5.749	F (4, 18) = 10.28	P=0.0002
met_species	49185	2	24593	F (2, 18) = 43970	P<0.0001
Tx	2.982e-010	2	1.491e-010	F (2, 18) = 2.666e-010	P>0.9999
Residual	10.07	18	0.5593		
Data summary					
Number of columns (Tx)	3				
Number of rows (met_species)	3				
Number of values	27				

2way ANOVA of 2PG:Multiple comparisons

	Data Set-A	Data Set-B	Data Set-C	Data Set-D	Data Set-E	Data Set-F	Data Set-G	Data Set-H
Within each row, compare columns (simple effects within rows)								
	3							
Number of families	3							
Number of comparisons per family	0.05							
Alpha								

Tukey's multiple comparisons test	Mean Diff.	95.00% CI of diff.	Below threshold?	Summary	Adjusted P Value			
m0								
UNT vs. ACT	2.173	0.6142 to 3.731	Yes	**	0.0061			
UNT vs. ACT+6AN	-0.3999	-1.958 to 1.159	No	ns	0.7920			
ACT vs. ACT+6AN	-2.573	-4.131 to -1.014	Yes	**	0.0014			
m1								
UNT vs. ACT	-2.173	-3.731 to -	Yes	**	0.0061			
UNT vs. ACT+6AN	0.3999	0.6142	No	ns	0.7920			
ACT vs. ACT+6AN	2.573	-1.159 to 1.958	Yes	**	0.0014			
		1.014 to 4.131						
m2								
UNT vs. ACT	0.000		No	ns	>0.9999			
UNT vs. ACT+6AN	0.000	-1.558 to 1.558	No	ns	>0.9999			
ACT vs. ACT+6AN	0.000	-1.558 to 1.558	No	ns	>0.9999			
		-1.558 to 1.558						
						N2	q	DF
Test details	Mean 1		Mean Diff.	SE of	N1			
		Mean 2		diff.				
m0						3	5.032	18.0
UNT vs. ACT	94.17		2.173		3	3	0.926	0
UNT vs. ACT+6AN	94.17	92.00	-0.3999	0.6106	3	3	2	18.0
ACT vs. ACT+6AN	92.00	94.57	-2.573	0.6106	3		5.958	0
		94.57		0.6106				18.0
m1						3		0
UNT vs. ACT	5.829		-2.173		3	3	5.032	
UNT vs. ACT+6AN	5.829	8.002	0.3999	0.6106	3	3	0.926	
ACT vs. ACT+6AN	8.002	5.430	2.573	0.6106	3		2	18.0
		5.430		0.6106			5.958	0
m2						3		18.0
UNT vs. ACT	0.000		0.000		3	3		0
UNT vs. ACT+6AN	0.000	0.000	0.000	0.6106	3	3	0.000	18.0
ACT vs. ACT+6AN	0.000	0.000	0.000	0.6106	3		0.000	0
		0.000		0.6106			0.000	
								18.0

								0<br>18.0<br>0<br>18.0<br>0

2way ANOVA of Lactate:ANOVA results

	Data Set-A	Data Set-B	Data Set-C	Data Set-D	Data Set-E
Table Analyzed	Lactate				
Two-way ANOVA	Ordinary				
Alpha	0.05				
Source of Variation	% of total variation	P value	P value summary	Significant?	
Interaction	1.878	<0.0001	****	Yes	
met_species	98.03	<0.0001	****	Yes	
Tx	3.275e-012	>0.9999	ns	No	
ANOVA table	SS	DF	MS	F (DFn, DFd)	P value
Interaction	582.4	6	97.07	F (6, 24) = 79.79	P<0.0001
met_species	30407	3	10136	F (3, 24) = 8331	P<0.0001
Tx	1.016e-009	2	5.079e-010	F (2, 24) = 4.175e-010	P>0.9999
Residual	29.20	24	1.217		
Data summary					
Number of columns (Tx)	3				
Number of rows (met_species)	4				
Number of values	36				

2way ANOVA of Lactate:Multiple comparisons

	Data Set-A	Data Set-B	Data Set-C	Data Set-D	Data Set-E	Data Set-F	Data Set-G	Data Set-H
Within each row, compare columns (simple effects within rows)								
	4							
Number of families	3							
Number of comparisons per family	0.05							
Alpha								
Tukey's multiple comparisons test	Mean Diff.	95.00% CI of diff.	Below threshold?	Summary	Adjusted P Value			
m0								
UNT vs. ACT	7.084	4.835 to 9.333	Yes	****	<0.0001			
UNT vs. ACT+6AN	-8.068	-10.32 to -5.819	Yes	****	<0.0001			
ACT vs. ACT+6AN	-15.15	-17.40 to -12.90	Yes	****	<0.0001			
m1								
UNT vs. ACT	-1.156	-3.405 to 1.093	No	ns	0.4180			
UNT vs. ACT+6AN	0.5559	-1.693 to 2.805	No	ns	0.8122			
ACT vs. ACT+6AN	1.712	-0.5375 to 3.961	No	ns	0.1603			
m2								
UNT vs. ACT	-5.630	-7.879 to -3.381	Yes	****	<0.0001			
UNT vs. ACT+6AN	6.770	4.521 to 9.019	Yes	****	<0.0001			
ACT vs. ACT+6AN	12.40	10.15 to 14.65	Yes	****	<0.0001			
m3								
UNT vs. ACT	-0.2985	-2.548 to 1.951	No	ns	0.9414			
UNT vs. ACT+6AN	0.7415	-1.508 to 2.991	No	ns	0.6925			
ACT vs. ACT+6AN	1.040	-1.209 to 3.289	No	ns	0.4908			
						N2	q	DF
Test details	Mean 1	Mean 2	Mean Diff.	SE of diff.	N1			
m0						3	11.12	24.0
UNT vs. ACT	72.78	65.69	7.084		3	3	12.67	0

UNT vs. ACT+6AN	72.78	80.84	-8.068	0.9006	3	3	23.79	24.0
ACT vs. ACT+6AN	65.69	80.84	-15.15	0.9006	3			0
				0.9006				24.0
m1						3	1.815	0
UNT vs. ACT	1.224	2.380	-1.156		3	3	0.872	
UNT vs. ACT+6AN	1.224	0.6683	0.5559	0.9006	3	3	9	
ACT vs. ACT+6AN	2.380	0.6683	1.712	0.9006	3		2.688	24.0
				0.9006				0
m2						3		24.0
UNT vs. ACT	23.31	28.94	-5.630		3	3	8.840	0
UNT vs. ACT+6AN	23.31	16.54	6.770	0.9006	3	3	10.63	24.0
ACT vs. ACT+6AN	28.94	16.54	12.40	0.9006	3		19.47	0
				0.9006				
m3						3		
UNT vs. ACT	2.688	2.987	-0.2985		3	3	0.468	24.0
UNT vs. ACT+6AN	2.688	1.947	0.7415	0.9006	3	3	8	0
ACT vs. ACT+6AN	2.987	1.947	1.040	0.9006	3		1.164	24.0
				0.9006			1.633	0
								24.0
								0
								24.0
								0
								24.0
								0
								24.0
								0

Ordinary one-way ANOVA of Pentose Cycle Activity:ANOVA results

	Data Set-A	Data Set-B	Data Set-C	Data Set-D	Data Set-E
Table Analyzed<br>Data sets analyzed<br><br>ANOVA summary	Pentose Cycle Activity<br>A-C				

F	29.04				
P value	0.0008				
P value summary	***				
Significant diff. among means (P < 0.05)?	Yes				
R squared	0.9064				
Brown-Forsythe test					
F (DFn, DFd)	0.1747 (2, 6)				
P value	0.8439				
P value summary	ns				
Are SDs significantly different (P < 0.05)?	No				
Bartlett's test					
Bartlett's statistic (corrected)					
P value					
P value summary					
Are SDs significantly different (P < 0.05)?					
ANOVA table	SS	DF	MS	F (DFn, DFd)	P value
Treatment (between columns)	2.813	2	1.406	F (2, 6) = 29.04	P=0.0008
Residual (within columns)	0.2906	6	0.04843		
Total	3.103	8			
Data summary					
Number of treatments (columns)	3				
Number of values (total)	9				

Ordinary one-way ANOVA of Pentose Cycle Activity:Multiple comparisons

	Data Set-A	Data Set-B	Data Set-C	Data Set-D	Data Set-E	Data Set-F	Data Set-G	Data Set-H
Number of families	1							
Number of comparisons per family	3							
Alpha	0.05							
Tukey's multiple comparisons test	Mean Diff.	95.00% CI of diff.	Below threshold?	Summary	Adjusted P Value			

UNT vs. ACT	-0.9556	-1.507 to -0.4042	Yes	**	0.0043	A-B		
UNT vs. ACT+6AN	0.3716	-0.1797 to 0.9230	No	ns	0.1770	A-C		
ACT vs. ACT+6AN	1.327	0.7759 to 1.879	Yes	***	0.0008	B-C		
Test details	Mean 1	Mean 2	Mean Diff.	SE of diff.	n1	n2	q	DF
UNT vs. ACT	1.710	2.665	-0.9556	0.1797	3	3	7.521	6
UNT vs. ACT+6AN	1.710	1.338	0.3716	0.1797	3	3	2.925	6
ACT vs. ACT+6AN	2.665	1.338	1.327	0.1797	3	3	10.45	6
Compact letter display								
ACT	A							
UNT	B							
ACT+6AN	B							

Ordinary one-way ANOVA of Pentose Cycle Activity:Descriptive statistics

	UNT	ACT	ACT+6AN
Number of values	3	3	3
Minimum	1.387	2.461	1.179
25% Percentile	1.387	2.461	1.179
Median	1.809	2.743	1.306
75% Percentile	1.934	2.792	1.529
Maximum	1.934	2.792	1.529
Mean	1.710	2.665	1.338
Std. Deviation	0.2866	0.1783	0.1771
Std. Error of Mean	0.1655	0.1030	0.1022
Lower 95% CI	0.9977	2.222	0.8982
Upper 95% CI	2.422	3.108	1.778

Supplemental Table 2. Single cell RNAseq data access and demographics.

GEO Sample Accession Number	Schafflick ID Code	Disease	Age	Sex
GSM4104122	MS19270	MS	35	F
GSM4104123	MS58637	MS	22	M
GSM4104124	MS71658	MS	47	F
GSM4104125	MS49131	MS	47	F
GSM4104126	MS60249	MS	28	F
GSM4104127	MS74594	MS	42	M
GSM4104128	PST83775	IIH	43	M
GSM4104129	PTC32190	IIH	33	M
GSM4104130	PST95809	IIH	43	F
GSM4104131	PTC41540	IIH	32	F
GSM4104132	PST45044	IIH	25	F
GSM4104133	PTC85037	IIH	25	F
