# Supplementary material for: Pentose phosphate pathway inhibition metabolically reprograms CD8+ T cells and disrupts CNS autoimmunity
Source: JCI Insight. 2025 Jun 10;10(14):e184240. doi: 10.1172/jci.insight.184240 (PMC12288970; doi:10.1172/jci.insight.184240)
Supplement: Unedited blot and gel images [file jciinsight-10-184240-s150.pdf]

Full uncropped western blots

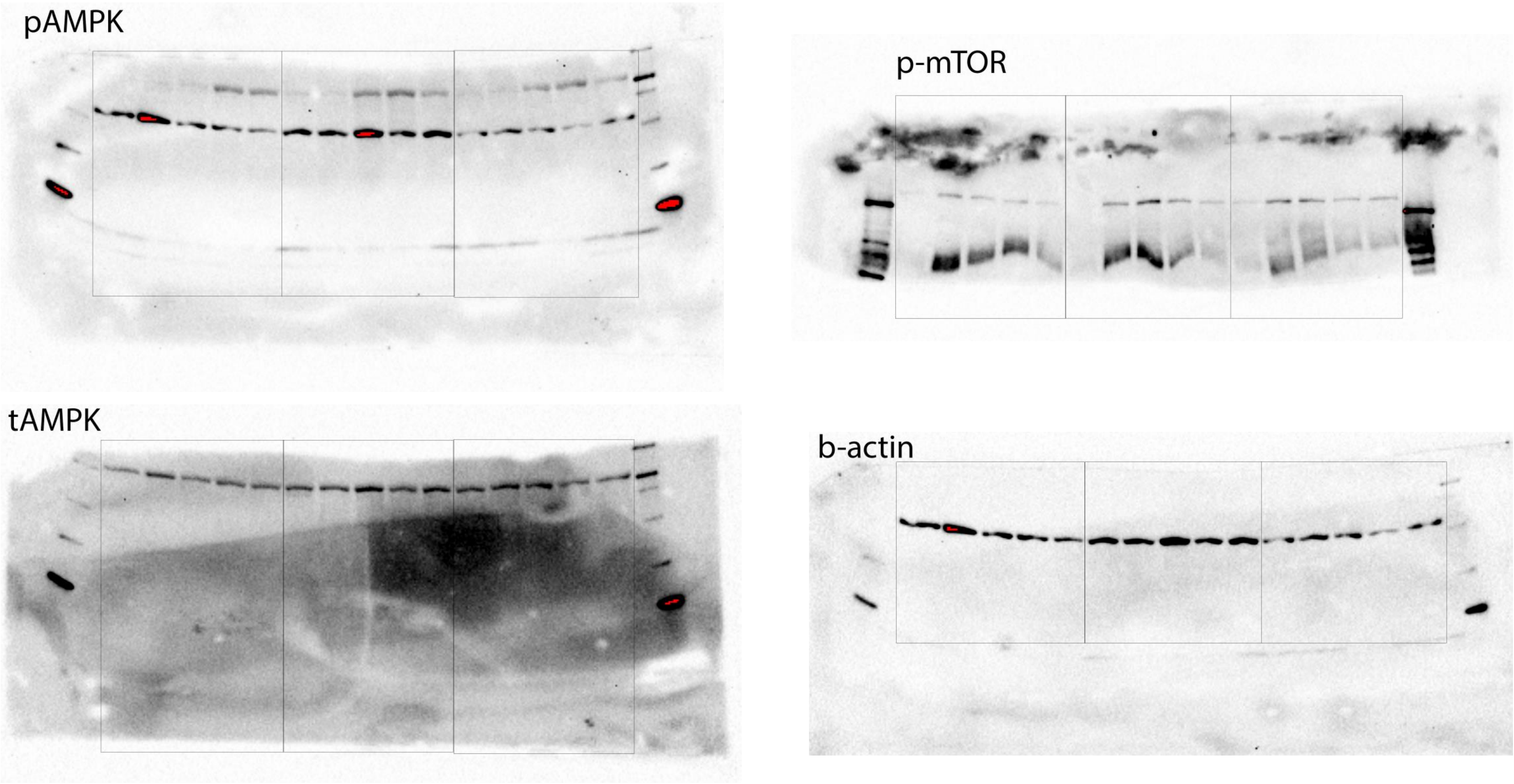

Boxes outline 5 samples per exp in 3 replicates
